# Discharge, Groundwater Gradients, and Streambed Micro‐Topography Control the Temporal Dynamics of Transient Storage in a Headwater Reach

**DOI:** 10.1029/2022WR034053

**Published:** 2023-07-10

**Authors:** Enrico Bonanno, Günter Blöschl, Julian Klaus

**Affiliations:** ^1^ Catchment and Eco‐Hydrology Group Luxembourg Institute of Science and Technology Belvaux Luxembourg; ^2^ Institute of Hydraulic and Water Resources Engineering Vienna University of Technology Vienna Austria; ^3^ Institute of Geography University of Bonn Bonn Germany

**Keywords:** transient storage, hyporheic zone, solute transport, groundwater/surface water interaction, stream tracer experiments, stream corridor

## Abstract

Contradictory interpretations of transient storage modeling (TSM) results of past studies hamper the understanding of how hydrologic conditions control solute transport in streams. To address this issue, we conduct 30 instantaneous tracer experiments in the Weierbach stream, Luxembourg. Using an iterative modeling approach, we calibrate TSM parameters and assess their identifiability across various hydrologic conditions. Near‐stream groundwater monitoring wells and LIDAR scans of the streambed are used to evaluate the area of the hyporheic zone and of the submerged sediments for each experiment. Our findings show that increasing discharge enhances parameters interaction requiring more samples of TSM parameters to obtain identifiable results. Our results also indicate that transient storage at the study site is influenced by in‐stream and hyporheic exchange processes during low discharge, likely due to the hyporheic zone's large extent and the relatively low water level compared to the size of slate fragments on the streambed. However, as discharge increases, in‐stream storage zones become part of the advective channel and the lower localized stream water losses to the adjacent groundwater suggests a decrease of the hyporheic exchange on transient storage. The results obtained were utilized to generate a hydrograph for the study site illustrating the dynamic evolution of in‐stream and hyporheic storage with varying discharge, providing insights into the expected influence of different transient storage processes prior to tracer experiments. Overall, our study enhances the understanding of the role of the hyporheic area and in‐stream storage zones in transient storage and helps estimate TSM parameters more accurately.

## Key Points

- Integrating groundwater data and streambed micro‐topography can improve the interpretation of transient storage model results
- Parameter interaction in transient storage models increases with discharge
- Hyporheic and in‐stream storage can both contribute to increase and decrease of stream water transient storage when discharges increase

## 1 Introduction

The understanding of how different hydrological processes control the transport of water downstream remains one of the most challenging topics in stream hydrology (Boano et al., 2014; Kelleher et al., 2019). The movement of water downstream is not only controlled by the advection‐dispersion process, but it is also governed by the transient storage process (Gooseff, Bencala, et al., 2008). Transient storage processes are the combined result of different mechanisms that delay the transport of water and solutes downstream compared to the advection‐dispersion processes. Transient storage can occur due to slowly moving surface water within the channel (i.e., in‐stream storage zones, Nordin & Troutman, 1980), due to the hydrologic exchange with subsurface water adjacent to and underlying the stream channel (i.e., the hyporheic zone, Bencala & Walters, 1983; Cardenas & Wilson, 2007), and due to the development of eddies and turbulences in the water column resulting from friction with in‐stream obstructions (Jackson et al., 2013). Despite the amount of work investigating the role of different processes on the transient storage of water in streams, the current state‐of‐the‐art is ambiguous on the understanding of how different hydrological processes and their interaction contribute to the transient storage across spatial scales and hydrologic conditions. Transient storage models (TSMs) are phenomenological models that provide a valuable tool to distinguish the role of advection‐dispersion and transient storage processes on water and solute transport in stream channels (Butturini & Sabater, 1999; Jin & Ward, 2005; Morrice et al., 1997; Zarnetske et al., 2007). However, in most studies the role of in‐stream storage zones and the hyporheic zone is inferred from TSM results only for a few hydrologic conditions and without information or data that can inform on the effective presence of transient storage zones (Runkel et al., 2003; Ward et al., 2019; Ward & Packman, 2019; Wondzell, 2006). These limitations hinder our ability to decipher how different parameters in the TSM influence the modeled BTC over the hydrological year and which hydrological process they indicate (Knapp & Kelleher, 2020).

The need to understand how different hydrological processes can influence water chemistry, biological activity, and the ecological richness of stream networks has motivated a range of studies over the past 30 years (Boulton et al., 2010; Stanford & Ward, 1988; Ward, 2016). Here, TSMs have been adopted to characterize hyporheic exchange and in‐stream water storage in a multitude of streams (Bencala et al., 2011; Bencala & Walters, 1983; Butturini & Sabater, 1999; Gooseff et al., 2005, 2008; Hart et al., 2002; Ward et al., 2018; Wörman, 1998). TSM assumes a uniform, steady‐state, 1‐D flow modeled via the advection‐dispersion equation (ADE) with a first‐order mass transfer exchange *α* between the advective flow channel and a finite‐sized storage zone with *A*_TS_ dimensions (Bencala & Walters, 1983). Knowing how the transient storage parameters *α* and *A*_TS_ change with different hydrologic conditions is crucial as larger transient storage zones and longer residence times are key factors in enhancing nutrient cycling (Argerich et al., 2011) and degrading pollutants (Moser et al., 2003) in stream networks. The simplified, yet informative structure of the TSM can thus offer valuable insights into the potential development of hot spots and hot moments that control water quality in surface waters (Krause et al., 2017; Smith, 2005).

Despite the pressing need to decipher the role of different hydrological processes and conditions on solute transport in streams, current research led to a collection of idiosyncratic studies with conflicting model predictions and interpretations (Ward & Packman, 2019). Previous studies have primarily investigated the variation of TSM parameters in terms of spatial relationships, interpreting changes in discharge as a function of increasing reach length or multiple adjacent reaches (Bencala & Walters, 1983; Bencala et al., 1990; D’Angelo et al., 1993; Scott et al., 2003; McKnight et al., 2004; Ward, Gooseff, et al., 2013; Gooseff et al., 2013). Alternatively, other studies have focused on changes in discharge and TSM parameters within the same stream reach (Edwardson et al., 2003; Jin & Ward, 2005; Mason et al., 2012; Schmid et al., 2010; Ward et al., 2018; Wlostowski et al., 2017). Despite the different approaches, no study has yet focused on the change of TSM parameters with discharge over the same stream reach for a wide range of hydrologic conditions (Table 1).

**Table 1:** Relationships Between Transient Storage Parameters *α* and *A*_TS_ and Stream Discharge *Q* in Published Literature

Study	Site name	Number and/or name of the sub‐reaches	Number of experiments per reach	*A*_TS_ versus *Q*	*α* versus *Q*	Fitting	Identifiability analysis
Legrand‐Marcq and Laudelout (1985)	Rieu d'Ostenne	1	13	(–)	Unclear	Visual	No
Bencala and Walters (1983)	Uvas Creek	5	1	(+)	Unclear	Visual	No
Bencala et al. (1990)	Snake river (upstream Deer Creek)	5	1	Unclear	Unclear	Visual	No
	Snake river (downstream Deer Creek)	3	1	Unclear	Unclear	Visual	No
D’Angelo et al. (1993)	Artificial streams	2 (Dogwood and Oak)	2 (summer‐winter)	/	/	Visual	No
	First‐order site	2 (Pine and Hardwood)	2 (summer‐winter)	(+)	(+)		
	Gradient site	4 (from first to fourth order)	1 (summer)	Unclear	Unclear		
	Fifth‐order site—unconstrained	2 (reaches 4 and 7)	2 (summer‐winter)	(–)	Unclear		
	Fifth‐order site—constrained	2 (reaches 1, 2, 3, 5, 6)	1 (summer)	Unclear	Unclear		
Valett et al. (1996)	Gallina Creek	1	4	(–)	/	Visual	No
Harvey et al. (1996)	St. Kevin Gulch	1	2	(–)	(+)	Nonlinear least squares regression	No
Morrice et al. (1997)	Gallina Creek	1	4	(–)	Unclear	Visual	No
Martí et al. (1997)	Sycamore Creek	1	8	(–)	Unclear	Visual	No
Butturini and Sabater (1999)	Riera Major stream	1	5	Unclear	Unclear	Direct fitting (sensu Hart, 1995)	No
Hart et al. (1999)	West Fork of Walker Branch	1	11 (^3^H) and 9 (Cl^−^)	Unclear	(+)	Direct fitting (sensu Hart, 1995)	No
Lees et al. (2000)	Mimram River	2	1	(+)	(+)	OTIS‐P (sensu Runkel, 1998)	Non‐unique convergence of *α* and *A*_TS_ parameters.
Hall et al. (2002)	Hubbard Brook Experimental Forest	1 (Bear brook)	4	Unclear	Unclear	Direct fitting (sensu Hart, 1995)	No
		1 (Cone Pond Outlet)	2	(+)	(+)		
		1 (Hubbard brook)	3	Unclear	Unclear		
		1 (Paradise brook)	2	(+)	(+)		
		W2 stream	3	(+)	Unclear		
		W3 stream	6	Unclear	Unclear		
		W4 stream	3	Unclear	Unclear		
		W5	3	(+)	Unclear		
		W6	5	Unclear	Unclear		
		West inlet to mirror lake	3	Unclear	Unclear		
Edwardson et al., 2003	Imnavait Creek	Site 1	2	(+)	(–)	OTIS‐P (sensu Runkel, 1998)	No
		Site 2	2	(–)	(+)		
	Blueberry Creek	Site 1	4	Unclear	(+)		
		Site 2	4	Unclear	Unclear		
	Toolik Inlet Stream	Site 1	2	(+)	(+)		
	Oksrukuyik Creek	Site 1	2	(–)	(+)		
	Kuparuk River	Site 1	6	Unclear	Unclear		
		Site 2	6	Unclear	Unclear		
Gooseff et al. (2003)	Lookout Creek watershed	LO411	2	(–)	(+)	UCODE plus manual modification of the parameters to visually match the tail of the BTC.	No
Scott et al. (2003)	Uvas Creek	5	1	(–)	Unclear	UCODE (unable to calibrate *α* and *A*_TS_ for two over five BTCs)	No
McKnight et al. (2004)	McMurdo Dry Valleys (Green Creek)	4	1	Unclear	Unclear	OTIS‐P (sensu Runkel, 1998)	No
Jin and Ward (2005)	Payne Creek	1	9 (constant‐rate)	Unclear	Unclear	OTIS‐P (sensu Runkel, 1998)	No
			6 (slug)	Unclear	Unclear		
Wondzell (2006)	WS1	1 (upper)	2	(–)	(+)	OTIS‐P (sensu Runkel, 1998)	No
		1 (lower)	2	(–)	(+)		
	WS3	1 (upper)	2	(–)	(–)		
		1 (lower)	2	(–)	(+)		
Zarnetske et al. (2007)	Northern foothills of Alaska's Brooks Range	1 (A2)	3	(+)	(+)	STAMMT‐L (sensu Haggerty et al., 2002)	No
		1 (P1)	4	Unclear	(+)		
		1 (AP)	4	Unclear	(+)		
Karwan and Saiers (2009)	Wangum Brook	1	3 (2)	(–)	(–)	Levenberg‐Marquardt nonlinear least squares algorithm (unable to calibrate *α* and *A*_TS_ under high discharge stages)	No
Schmid et al. (2010)	Mödlingbach	1	12	(+)	(+)	OTIS‐P (sensu Runkel, 1998)	No
	Torrente Lura	S‐I	4	(+)	(+)		
		S‐II	7	(+)	(+)		
		D‐G	5	(+)	(+)		
Fabian et al. (2011)	Prieta Creek	1	3	Unclear	(+)	OTIS‐P (sensu Runkel, 1998)	No
Mason et al. (2012)	Silver Bow Creek	1	58	/	/	/	Identifiability analysis—*α* and *A*_TS_ non‐identifiable
Ward, Gooseff, et al. (2013)	Stringer Creek	28	4	Unclear	Unclear	OTIS + Monte Carlo simulations (100,000)	Identifiability analysis—*α* and *A*_TS_ non‐identifiable
Gooseff et al. (2013)	Uvas Creek	5 (+10 sub‐reach combinations)	1	(+)	Unclear	OTIS + UCODE (sensu Scott et al., 2003)	No (suspected non‐identifiability for some results)
González‐pinzón et al. (2015)	Shaver Creek	2	1	(+)	(–)	OTIS + Shuffled Complex Evolutionary algorithm	No
Wlostowski et al. (2017)	Alaska's North Slope	I8 inlet	4	Unclear	Unclear	OTIS + Shuffled Complex Evolutionary algorithm	No
		Peat inlet	3	(+)	(+)		
Ward et al. (2017)	Tenderfoot Creek Experimental Forest	2 (100–2,500 m)	1	(–)	(–)	OTIS + Monte Carlo simulations (100,000)	Identifiability analysis—*D*, *A*, *α* and *A*_TS_ non‐identifiable
Ward et al. (2018)	Fawn River	Unrestored reach	4	(+)	(+)	OTIS + Monte Carlo simulations (100,000)	Identifiability analysis—*D*, *A*, *α* and *A*_TS_ identifiable

*Note.* (+) and (−) symbols indicate respectively positive and negative relationships between stream discharge with the transient storage parameters *α* and *A*_TS_. The “unclear” term means that both positive and negative relationships have been observed between discharge and *α* and *A*_TS_. The slash symbol (/) indicates no results, due to non‐reported results or a null evaluation of the parameter. When a study investigated multiple stream reaches, we reported only the reaches investigated at different hydrologic conditions. We here also reported studies using only one tracer injection, but where higher discharge was studied via multiple reaches with tracer measurement location located at increasing distance from the tracer injection point.

Higher discharge has been linked to higher (Bencala & Walters, 1983; Lees et al., 2000; Schmid et al., 2010; Ward et al., 2018; Wlostowski et al., 2013) and lower (Harvey et al., 1996; Karwan & Saiers, 2009; Martí et al., 1997; Morrice et al., 1997; Valett et al., 1996; Ward et al., 2017; Wondzell, 2006) values of the transient storage area *A*_TS_, or to show no clear relationships (Edwardson et al., 2003; Fabian et al., 2011; Hall et al., 2002; Jin & Ward, 2005; McKnight et al., 2004; Ward, Payn, et al., 2013; Zarnetske et al., 2007). Similarly, the rate of exchange *α* between the advective flow channel with the transient storage zone was higher (Fabian et al., 2011; Gooseff et al., 2003; Hart et al., 1999; Harvey et al., 1996; Lees et al., 2000; Schmid et al., 2010; Ward et al., 2018; Wondzell, 2006), lower (González‐pinzón et al., 2015; Karwan & Saiers, 2009; Ward et al., 2017), or showed no clear relationship with discharge (Bencala et al., 1990; Bencala & Walters, 1983; Butturini & Sabater, 1999; D’Angelo et al., 1993; Edwardson et al., 2003; Gooseff et al., 2013; Hall et al., 2002; Jin & Ward, 2005; Legrand‐Marcq & Laudelout, 1985; Martí et al., 1997; McKnight et al., 2004; Morrice et al., 1997; Scott et al., 2003; Ward, Payn, et al., 2013).

The inconsistency in the relation between the values of TSM parameters with discharge in previous studies (Table 1) might derive from the specific characteristics of the different study sites. For example, stream channels with relatively high hydraulic conductivity of the streambed material (sand and gravel) would allow the inflow of water from the stream toward the adjacent groundwater with higher discharge (Dudley‐Southern & Binley, 2015), leading to a more pronounced tail of the BTC (Schmadel et al., 2016). In contrast, stream channels confined by fresh bedrock or characterized by material with low hydraulic conductivity would exhibit a rather reduced hyporheic zone area at high discharge and are thus more likely to show a reduction in transient storage area with higher discharge (Wondzell, 2011). Nevertheless, conflicting or absent relationships between TSM parameters and stream discharge could also be due to limitations that are common in studies investigating solute transport in streams with tracer experiments (Table 1). First, the calibration of parameters in TSM has been performed iteratively to visually fit the modeled BTC over the observed BTC (Bencala et al., 1990; Bencala & Walters, 1983; D’Angelo et al., 1993; Legrand‐Marcq & Laudelout, 1985; Valett et al., 1996), or by inverse modeling (Edwardson et al., 2003; Fabian et al., 2011; Gooseff et al., 2013; Jin & Ward, 2005; Lees et al., 2000; McKnight et al., 2004; Schmid et al., 2010; Wondzell, 2006). However, the identifiability of model parameters has generally not been taken into account in most TSM studies (Table 1), leading to a lack of certainty about the modeling results and their physical interpretation (Knapp & Kelleher, 2020). When TSM parameters are non‐identifiable, they are highly interdependent, meaning that changes in one parameter would be balanced by a proportional change of one or more other parameters leading to the same model performances (Camacho & González, 2008; Kelleher et al., 2013; Wagener, Lees, & Wheater, 2002; Wagner & Harvey, 1997). Identifiability is a crucial issue for the interpretation of TSM results, as most studies that have addressed the identifiability of TSM parameters have found that they were non‐identifiable (Camacho & González, 2008; Kelleher et al., 2019; Wagener, Lees, & Wheater, 2002; Ward, Kelleher, et al., 2017; Wlostowski et al., 2013). Non‐identifiability of TSM parameters does not only affect model performance, but it can result in the modeled BTC mimicking the advection‐dispersion equation, leading to a misinterpretation of the processes governing transient storage at the study site (Bonanno, Blöschl, & Klaus, 2022). A growing number of studies addressed parameter identifiability in TSMs via random sampling approaches (Kelleher et al., 2019; Ward et al., 2017, 2018; Knapp & Kelleher, 2020; Table 1). However, no study to date has directly investigated the identifiability of the TSM parameters under multiple hydrologic conditions, which may improve our understanding of why TSM parameters were identifiable in some studies (Ward et al., 2017, 2018) and not in others (Camacho & González, 2008; Kelleher et al., 2013; Wagener, Lees, & Wheater, 2002; Ward, Kelleher, et al., 2017; Wlostowski et al., 2013).

There is also a second limitation that may cause an unclear correlation between TSM parameters and discharge, thus hindering the physical interpretation of the model results, namely the scarcity of information about the stream reach and the relatively small number of tracer experiments. Studies analyzing a section of a stream under different hydrological conditions, or studying higher stream discharge at successive monitoring stations, rarely investigated more than four stages of discharge (Table 1) with the tracer experiments being mostly conducted at baseflow conditions (Ward, 2016). A relatively low number of investigated discharge stages hampers the ability to observe a robust relationship between discharge and *α* and *A*_TS_, resulting in a poor understanding of the processes controlling transient storage in stream reaches (Ward & Packman, 2019). Investigating the link between TSM parameters across different discharge stages is also not enough for associating specific hydrological processes with certain hydrologic conditions. This is because higher discharge can cause larger and lower hyporheic exchange and in‐stream transient storage depending on the stream morphology and the groundwater gradients at the study site (Bonanno et al., 2021; Dudley‐Southern & Binley, 2015; Gooseff, Bencala, et al., 2008; Jin & Ward, 2005; Martí et al., 1997; Schmid et al., 2010). A clearer perception of the physical processes of stream reaches fosters a robust interpretation of TSM results. As an example, the measurements of the groundwater levels adjacent to the stream channel can be used to infer if the near‐stream groundwater is receiving stream water, thus offering valuable information on the potential development of hyporheic zones at the study site (Bonanno et al., 2021; Voltz et al., 2013; Wondzell, 2006). Likewise, the size and distribution of the sediments making the streambed topography can be a crucial resource for the interpretation of TSM results, since streambed sediments and in‐stream obstructions can create recirculating zones resulting in a non‐negligible role for in‐stream transient storage (Hart et al., 1999; Jackson et al., 2013; Montgomery & Buffington, 1997). Without a comprehensive knowledge of the groundwater levels and the streambed topography, it is difficult to distinguish whether a certain transient storage area can be related to a specific transient storage process or whether it is simply the result of a mathematical fit of the TSM parameters without a realistic role for solute transport at the study site.

In this manuscript we address the following research questions.

1. Does the identifiability of TSM parameters change with discharge?
2. How do transient storage processes change under different hydrologic conditions?

To answer these questions and to overcome the limitations mentioned above in TSM studies we performed 30 in‐stream tracer experiments and we: (a) investigated the identifiability of TSM parameters by combining global identifiability analysis with dynamic identifiability analysis in an iterative approach obtaining identifiable TSM parameters (Bonanno, 2022; Bonanno, Blöschl, & Klaus, 2022); (b) we recorded the groundwater elevation at the study site through a groundwater monitoring network of 43 wells that allowed us to infer the extent of the hyporheic zone during each experiment; (c) we obtained the micro‐topography of the streambed via a laser scan and we compared the distribution of the height of the slate fragments in the streambed to the surface water level to infer the role of in‐stream transient storage in different hydrologic conditions.

## 2 Methods

### 2.1 Study Site

**Figure 1:**
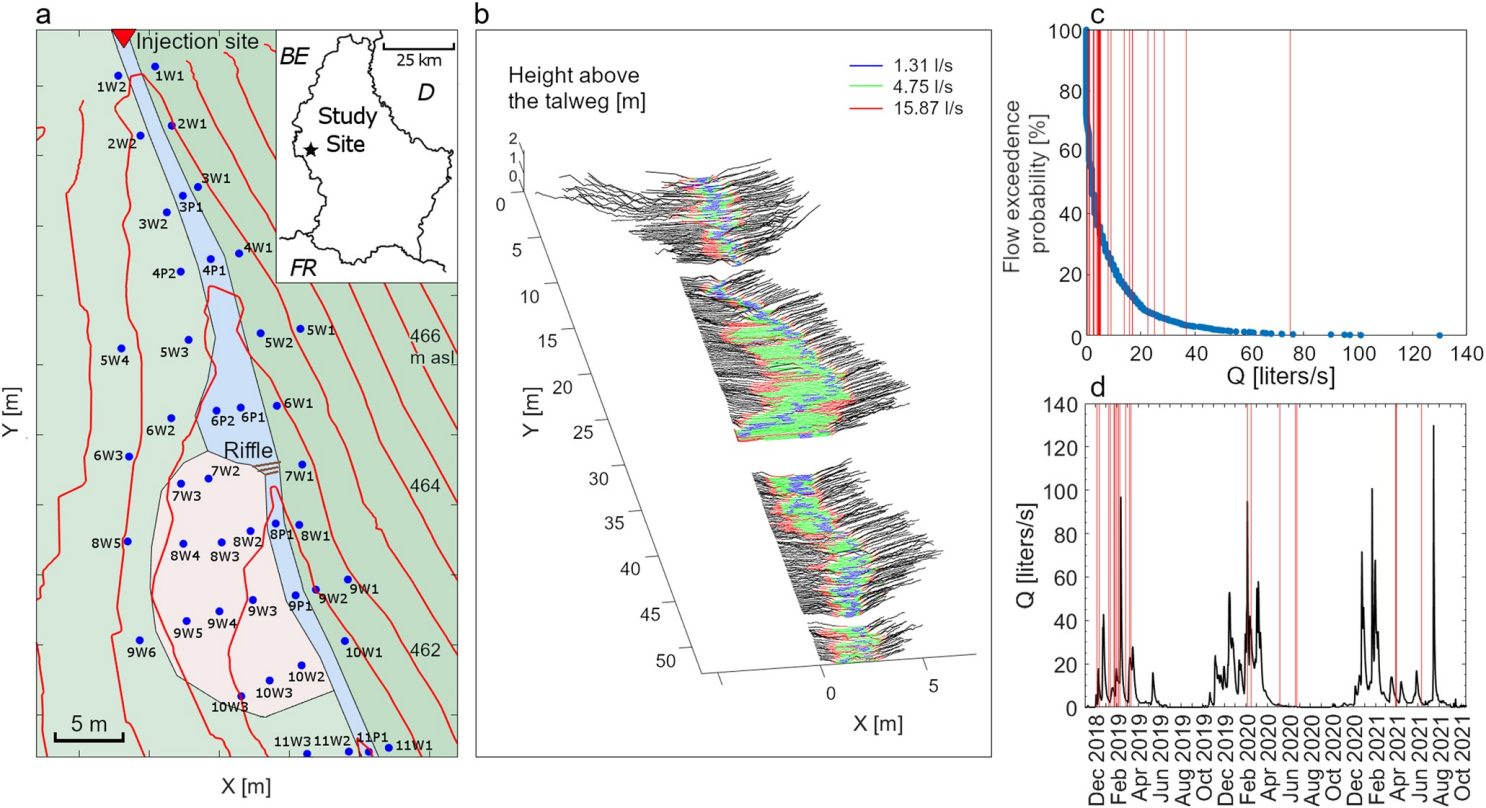
(a) Study reach and location of the wells and piezometers. The *Y* direction corresponds to the direction of the geographic north; thus the stream reach defines an “east footslope” on the right side of the map, and a “west footslope,” on the left side of the map. (b) Transects of the stream channel were extracted from four LIDAR scans at a 1 cm resolution and 15 cm distance between each other. The white areas between the scans are missing data due to the local presence of water and shadow zones. Blue, green, and red lines indicate the length of the wetted perimeter in each transect for the three reported discharge stages. (c) Flow duration curve for the recorded discharge at the Weierbach outlet for the period January 2018—December 2021, 55 m upstream of the study site. (d) Hydrograph of the Weierbach outlet for the investigation period. The vertical red lines in (c) and (d) indicate the discharge stages investigated during the slug tracer injections.

The study reach is located in western Luxembourg, downstream of the Weierbach experimental catchment (49°49′38″N, 5°47′44″E) (Hissler et al., 2021). The stream reach is 55 m long and it is characterized by a riffle morphology, has an average slope of ≃6%, is unvegetated, and consists of deposited colluvium of fragmented slates over a fractured bedrock layer (Bonanno et al., 2021; Figure 1). Previous work outlined the occurrence of several hydrological processes controlling stream water generation in the Weierbach catchment. The hillslopes at the study site are characterized by a regolith layer with a relatively high hydraulic conductivity compared to the fractured bedrock layer beneath (Glaser et al., 2016, 2020). The subsurface structure does not promote shallow lateral flow toward the stream channel (Klaus & Jackson, 2018), and precipitation water percolates vertically toward the groundwater table in the fractured bedrock (Rodriguez & Klaus, 2019). The water movement through and above the fractured bedrock from the hillslopes maintains a rather steady and shallow groundwater level in the near‐stream domain throughout the year (Fabiani et al., 2021), and the organic soil areas composing part of the riparian area are almost constantly saturated (Bonanno et al., 2021). Discharge is thus generated by both a fast‐ and a slow‐response to precipitation events. The fast‐response is controlled by the surface runoff of event‐water over the saturated organic soil in the riparian zone toward the stream channel (Antonelli et al., 2020a, 2020b; Bonanno et al., 2021; Wrede et al., 2015). The slow‐response occurs when the amount of water from precipitation events exceeds the storage capacity at the hillslope. When this happens, the groundwater is laterally redistributed over the fractured bedrock from the hillslopes toward the stream channel causing an increase in discharge and a double‐peak behavior in the hydrograph (Bonanno et al., 2021; Martínez‐Carreras et al., 2016).

### 2.2 Tracer Experiments

We performed a total of 30 in‐stream instantaneous tracer experiments between December 2018 and June 2021. For each experiment, we prepared a NaCl solution using 2 L of stream water and an amount of NaCl between 50 and 250 g (Table 2). We selected slug injections over constant‐rate injections to minimize the influence of varying discharge on the BTC measurements (Ward, Gooseff, et al., 2013) and because they contain the same information as a constant‐rate injection for conservative tracers (Payn et al., 2008). We injected the tracer solution in a turbulent pool at the beginning of the study reach, assuring complete mixing. We measured electric conductivity (EC) at the end of the investigated reach (55 m from the injection point) via a portable conductivity meter (WTW TetraCon 3310) providing a resolution of 0.1 µS/cm from 0 to 199 µS/cm and 1 µS/cm from 200 to 1,999 µS/cm (accuracy ± 0.5% of the value and temperature automatically compensated). Calibration of EC to chloride concentration was conducted in the laboratory using a known volume of water sampled at the measurement location before the tracer injection. The conversion into chloride concentration was obtained via an EC‐Cl^−^ regression line (*R* ^2^ > 0.99) and the background concentration was removed using water samples taken before and after the end of the experiment at the injection point. Discharge (*Q* [L^3^/T]) during each tracer injection was evaluated using the recorded BTC via the dilution gauging method (Beven et al., 1979; Butterworth et al., 2000). The experiments were carried out across different discharge stages of the Weierbach over three hydrologic years (October 2018—October 2021, Table 2; Figures 1c and 1d. BTC data available in Bonanno, Barnich, et al., 2022).

**Table 2:** List of the In‐Stream Instantaneous Tracer Injections, Date, Discharge From Dilution Gauging, and Amount of Injected NaCl

ID number	Date	Discharge *Q* (liters/s)	Amount of NaCl (g)
E01	6 December 2018	2.5	100
E02	11 December 2018	14.0	100
E03	8 January 2019	4.5	100
E04	11 January 2019	3.8	100
E05	23 January 2019	9.0	100
E06	24 January 2019	7.9	100
E07	28 January 2019	22.8	100
E08	4 February 2019	17.0	100
E09	5 February 2019	17.1	100
E10	8 February 2019	15.9	100
E11	25 February 2019a	5.3	100
E12	25 February 2019b	4.9	100
E13	25 February 2019c	4.7	100
E14	8 March 2019	28.6	100
E15	11 March 2019	25.2	100
E16	5 February 2020	75.0	100
E17	14 February 2020	36.7	250
E18	6 May 2020	1.3	150
E19	18 June 2020	0.9	100
E20	22 June 2020	0.4	100
E21	29 March 2021a	5.1	100
E22	29 March 2021b	5.0	100
E23	30 March 2021a	4.8	100
E24	30 March 2021b	4.7	100
E25	31 March 2021a	4.7	50
E26	31 March 2021b	4.6	100
E27	31 March 2021c	4.6	150
E28	31 March 2021d	4.1	200
E29	11 June 2021a	2.8	100
E30	11 June 2021b	2.5	100

### 2.3 Water Table Measurements and Groundwater Flow Direction

The study site is equipped with a network of 36 wells and seven piezometers (Figure 1a). The wells are in the near‐stream domain, while the piezometers are located in the streambed (Bonanno et al., 2021). We manually measured the water level in the monitoring network using a water level meter (Eijkelkamp 11.03) immediately before the injection of the tracers into the stream channel. The piezometers in the stream channel allowed us to measure both the groundwater hydraulic head and water depth in the stream channel.

We obtained the groundwater table at the study site by interpolating the measured groundwater level in the network via spline interpolation (Matlab R2020a, The Mathworks, Natick, MA). The interpolated groundwater table was used to evaluate the groundwater flow direction in the area between the wells in the monitoring network during each instantaneous tracer injection. If the extracted flow direction was pointing away or toward the stream channel, then that stream section was considered in gaining and losing condition, respectively (Bonanno et al., 2021). The area with the groundwater flow direction pointing away from the stream channel and then returning to the stream channel was considered as the size of the hyporheic zone on the *xy* plane at the investigated hydrologic conditions. The corresponding volume of the hyporheic zone was approximated as equal to the hyporheic zone area on the *xy* plane multiplied by the average water depth in the stream channel. The volume of the hyporheic zone so obtained was then averaged over the reach length (55 m), to estimate the average area of the hyporheic zone on the plane perpendicular to the stream reach. It is important to note that, compared to the hyporheic zone extension on the *xy* plane, the area of the hyporheic zone averaged on the reach length was not directly measured. However, this quantity is particularly valuable because it allows for a direct comparison with the area of the transient storage as measured by the TSM, since both of them are a measure of the transient storage zone area perpendicular to the flow direction (Runkel et al., 2003).

### 2.4 Evaluation of Streambed Micro‐Topography

We conducted four Laser Imaging Detection and Ranging (LIDAR) scans at the study site via terrestrial Faro Photon 120/20 (accuracy ± 2 mm at 25 m) prior to the tracer experiments (20 November 2018 to evaluate the size and the spatial distribution of the fragmented slates over the fractured bedrock in the streambed. Each scan was analyzed via Cloud Compare (version 2.11.3, 2020). The stream reach has been subdivided into transects with a 1 cm resolution, perpendicular to the stream talweg and distanced 15 cm from each other (black lines, Figure 1b). We evaluated the size and distribution of the slate fragments above the talweg for each LIDAR stream channel section. Knowing the water level recorded in the stream channel, each transect has also been used to evaluate the length of the wetted perimeter for each water level (blue, green and red lines, Figure 1b). We used the calculated wetted perimeter and the successive evaluation of the wetted stream area via TSM to evaluate the average hydraulic radius (*H*_*R*_) during each tracer injection.

### 2.5 Formulation of the Transient Storage Model

The formulation of the TSM reads (Bencala & Walters, 1983):

(1) $$\left\{\begin{array}{c} \frac{\partial C}{\partial t}=-v\frac{\partial C}{\partial x}+\frac{1}{A}\frac{\partial }{\partial x}\left(AD\frac{\partial C}{\partial x}\right)+\alpha \left(C_{TS}-C\right)\\ \frac{\partial C_{TS}}{\partial t}=-\alpha \frac{A}{A_{TS}}\left(C_{TS}-C\right) \end{array}\right.$$

where *t* is time [T], *x* is the distance from the injection point along the stream reach [L], *A* [L^2^] is the stream discharge cross‐sectional area, *v* [L/T] is the flow velocity, *D* [L^2^/T] is the longitudinal dispersion coefficient, *C* and *C*_TS_ are the concentration of the observed tracer in the stream channel and in the storage zone, respectively [M/L^3^], *α* [1/T] is the exchange coefficient between the stream channel and the storage zone and *A*_TS_ [L^2^] is the area of the transient storage zone. Three primary assumptions are associated with the formulation of the TSM (Harvey et al., 1996): (a) negligible transport in the hyporheic zone parallel to the stream, (b) exponential residence time distribution (RTD) in the transient storage zone, and (c) in‐stream water storage and hyporheic exchange are jointly described by the transient storage parameters *α* and *A*_TS_. This last limitation reflects the lumped nature of the single storage zone described in the TSM which is thus not able to distinguish between the cross‐sectional area of in‐stream storage zones and the hyporheic zone (Runkel et al., 2003).

### 2.6 Calibration and Identifiability of Transient Storage Model Parameters

#### 2.6.1 Identifiable and Non‐Identifiable TSM Parameters

TSM parameters are usually obtained via visual fitting or inverse modeling approaches, such as OTIS‐P (Table 1). Despite the good model performances that can be obtained from inverse modeling approaches, the parameters might be non‐identifiable (Kelleher et al., 2019) and may not capture the underlying processes well. Thus, several authors advocated the identification of a “behavioral” parameter population in TSMs via identifiability analysis (i.e., parameter sets satisfying certain performance thresholds) since this is a preferable and more informative outcome than a singular best set of parameter values (Beven, 2001; Kelleher et al., 2019; Wagener, Lees, & Wheater, 2002; Wagener et al., 2002, 2002; Wlostowski et al., 2013).

The “identifiability” term is used to indicate how certain a parameter is in a model application. If a good model performance occurs in only a relatively narrow parameter interval compared to the distribution of its possible values, then the parameter can be considered identifiable. On the contrary, if a good model performance is distributed across a relatively large parameter interval compared to the distribution of its possible values, then the parameter can be considered non‐identifiable (Ward et al., 2017). In studies where the identifiability of TSM parameters has been investigated via random sampling approaches, *α* and *A*_TS_ have proved to be rarely identifiable (Kelleher et al., 2013; Ward, Gooseff, et al., 2013, 2017).

#### 2.6.2 The GlaDy Identifiability Analysis for TSM

We obtained TSM parameters and their identifiability via a novel iterative modeling approach that combines random sampling with Global identifiability analysis and Dynamic identifiability analysis (GlaDy, Bonanno, 2022). The GlaDy identifiability analysis requires a certain number of parameter sets and the corresponding model performance compared to the observed BTC. By “parameter set” we refer to a specific combination of *v*, *A*, *D*, *α*, and *A*_TS_ parameter values, while by “parameter space” we refer to the range of a parameter between the selected lower and the upper bounds in the random parameter sampling. Model performance was evaluated with the Nash‐Sutcliffe objective function (NSE). We selected NSE because it allows consistency between the dynamic identifiability analysis and global identifiability analysis used in the iterative modeling approach (Bonanno, Blöschl, & Klaus, 2022; Wagener, Lees, & Wheater, 2002). Additionally, NSE is a normalized objective function that is beneficial for comparing performances across runs and among tracer experiments. Global identifiability analysis addresses the identifiability of the TSM parameters using as model performances the NSE evaluated on the entire BTC, while the dynamic identifiability analysis addresses the identifiability of the TSM parameters over time, meaning that identifiability is studied along a moving window over the BTC (Wagener, Lees, & Wheater, 2002).

Compared to other work addressing the identifiability of TSM we here considered as a calibration parameter also the flow velocity, *v*. This is because it was proven that keeping *v* as a constant model parameter might cause misestimation of the other TSM parameters during identifiability analysis (Bonanno, Blöschl, & Klaus, 2022). By considering *v* as a calibration parameter we also avoid the uncertainty inevitably bonded to experimental velocity measurements or the discharge evaluation via dilution gauging method, since *Q = v∙A* (Schmadel et al., 2010).

Before the application of the TSM, we simulated every tracer experiment via the classic advection‐dispersion equation (ADE) to avoid any initial assumptions of the advection‐dispersion parameters (*v*, *A*, *D*) that might affect the results of the iterative modeling approach. 35,000 ADE parameter sets have been uniformly sampled from the feasible parameter space (see Bonanno, Blöschl, & Klaus, 2022) and the parameter set with the highest NSE defined the optimal ADE fitting with parameters *v*_ADE_, *A*_ADE_, *D*_ADE_, and performance NSE_ADE_.

Starting from ADE results (*v*_ADE_, *A*_ADE_, *D*_ADE_) the GlaDy identifiability analysis conduces a first iteration by randomly sampling via Latin Hypercube sampling 35,000 TSM parameters set (*v*, *A*, *D*, *α*, and *A*_TS_) over a relatively large parameter space (Table 3) and evaluating the corresponding model performance using OTIS as a solver. This number of parameter sets was chosen for every TSM iteration because 35,000 parameter sets were proven to have always less than 2% error in the mean and standard deviation of the top 10% results compared to 115,000 parameter sets, when the identifiability conditions are met (Bonanno, Blöschl, & Klaus, 2022). The upstream boundary condition was set as instantaneous (IBOUND = 1, in OTIS) over three time steps: at zero and 2 s, the concentration was set to zero, while at one second, the concentration was calculated as the mass of the solute divided by the product of the randomly sampled velocity and area, multiplied by the time interval used for the injection (1 s). As a result, each randomly sampled parameter set also considered its specific concentration as the upstream boundary condition.

**Table 3:** List of Calibration Parameters and Used Initial Ranges for the First Iteration of the GlaDy Identifiability Analysis

Abbrev.	Parameter	Lower bound	Upper bound
*v* [m/s]	Flow velocity	0.5 ·*v*_ADE_	2 ·*v*_ADE_
*A* [m^2^]	Advective channel cross‐sectional area	0.5 ·*A*_ADE_	2 ·*A*_ADE_
*D* [m^2^/s]	Longitudinal dispersion coefficient	10^–4^	2 ·*D*_ADE_
*α* [s^−1^]	Transient storage exchange rate	10^–5^	0.1
*A*_TS_ [m^2^]	Transient storage cross‐sectional area	10^–5^	1

*Note.* The subscript ADE indicates the best‐fitting model parameter obtained after the BTC fitting using the advection‐dispersion equation (cfr Section 2.6.2).

The GlaDy identifiability analysis is iterative, meaning that a successive TSM iteration depends on the results of the identifiability analysis of the previous iteration. This is similar to previous studies that used a random sampling approach combined with behavioral thresholds (Kelleher et al., 2013; Ward, Kelleher, et al., 2017). The following TSM iterations rely on a constrained parameter space depending on the best‐performing upper and lower bound obtained by the results of the global and dynamic identifiability analysis.

#### 2.6.3 Conditions for Global and Dynamic Identifiability

Globally identifiable parameters have: (a) univocal peak of performance in parameter versus objective function plots (Ward et al., 2017); (b) cumulative distribution function (CDF) corresponding to the best 0.1% of the model results deviating from the 1:1 line and from parameter CDF corresponding to the best 10% of the model results (Kelleher et al., 2019); (c) the two‐sample Kolmogorov‐Smirnov (K‐S) test indicating a statistically relevant difference in the CDF corresponding to the best 0.1% and 10% results (*p* ≤ 0.05, Bonanno, Blöschl, & Klaus, 2022):

(2) $$\left[K,p\right]=\max\left|F\left(P_{0.1}\right)-F\left(P_{10}\right)\right|$$

where *F*(*P*_0.1_) and *F*(*P*_10_) are the cumulative distribution function of a parameter *P* respectively for the best 0.1% and the best 10% of the model results. Dynamic identifiability analysis indicates the 90% confidence interval of a parameter compared to the considered parameter space over different sections of the BTC. The evaluation of one minus the width of the 90% confidence interval over the entire parameter range indicates the “information content” of a certain parameter over the BTC. Information content values close to one indicate stronger parameter identifiability in that investigated section of the BTC compared to lower information content values (Bonanno, Blöschl, & Klaus, 2022; Wagener, Lees, & Wheater, 2002).

#### 2.6.4 Dependency of Identifiability of TSM Parameters With the Number of Iterations

The GlaDy identifiability analysis was finalized once all model parameters (*v*, *A*, *D*, *α*, and *A*_TS_) satisfied the global identifiability conditions defined above in their selected parameter space. We repeated the sampling of 35,000 parameter sets over the parameter space that indicated identifiability until we obtained at least 1,000 parameter sets that perform better than the ADE (NSE > NSE_ADE_). This was done to obtain a statistically relevant number of parameter sets with satisfactory model performances because only TSM parameter sets with better performances than the ADE should be used for the interpretation of model results (Bonanno, Blöschl, & Klaus, 2022). The successive analysis of the transient storage process and transport metrics were conducted only on the behavioral parameter sets (NSE > NSE_ADE_).

After every model iteration, we evaluated the mean and standard deviation of the top 10% NSE for all the modeling results and behavioral parameter sets only. This is because the top 10% of the results are often used as a behavioral threshold in several studies addressing the identifiability of TSM parameters (Kelleher et al., 2019; Wagener, Lees, & Wheater, 2002; Ward, Kelleher, et al., 2017). Following Pianosi et al. (2015) and Bonanno, Blöschl, and Klaus (2022), we interpreted a decrease in the mean and standard deviation of NSE with an increasing number of TSM iterations as an increase in model identifiability. On the contrary, the constant mean and standard deviation of NSE with an increasing number of iterations have been interpreted as the model is unable to increase TSM performances with increasing iterations.

#### 2.6.5 Comparison With OTIS‐P Modeling Approach

We here compared our results to the more traditional inverse modeling approach, OTIS‐P. This is because when OTIS‐P is used, no identifiability study is generally conducted to address the reliability of the TSM parameters (Fabian et al., 2011; Jin & Ward, 2005; McKnight et al., 2004; Schmid et al., 2010) while only a few studies compared random sampling approach with OTIS‐P (Ward et al., 2017, 2018). By comparing the GlaDy identifiability analysis introduced in Bonanno, Blöschl, and Klaus (2022) with OTIS‐P we aim for a more robust assessment of parameter estimation and their identifiability not previously reported in the literature.

OTIS‐P uses a non‐linear regression scheme to minimize the residual sum of squares between the modeled BTC and the observed BTC and returns the 95% confidence interval for the estimated TSM parameters. Following Runkel (1998), we carried out multiple OTIS‐P iterations starting from different initial parameter values (*A* = *A*_ADE_, *D* = *D*_ADE_ and *α* = 0.1 s^−1^, *A*_TS_ = 0.1 m^2^; *α* = 0.01 s^−1^, *A*_TS_ = 0.01 m^2^; *α* = 0.001 s^−1^, *A*_TS_ = 0.001 m^2^). This was done to avoid false model convergence to a local minimum. We applied OTIS‐P in consecutive steps, setting the results obtained from the previous modeling output as starting parameter values of the successive software application. We finalized the use of OTIS‐P when parameter values changed less than 0.1% between subsequent runs (Ward et al., 2017). When OTIS‐P was not able to converge to a unique set of parameter values or indicated convergence errors, we discarded its results.

### 2.7 Metrics Characterizing Solute Transport in Stream

We computed several metrics from the best‐performing parameter sets (NSE > NSE_ADE_) related to solute transport and storage in the study reach.

We evaluated the average residence time of a tracer molecule in the transient storage zone (*RT*_s_ [T]) and the average time a tracer molecule remains in the stream channel before passing into the storage zone (*RT*_Q_ [T]) (Runkel, 2002; Thackston & Schnelle, 1970):

(3) $${RT}_{Q}=\frac{1}{\alpha }$$

(4) $${RT}_{S}=\frac{A_{TS}}{\alpha \cdot A}$$

We obtained the total water flux exchanged between the stream channel and the storage zone. This was done by multiplying the average water flux through the storage zone per unit length of the stream channel by the reach length *L* (*q*_*s*_ [L^3^/T], modified from Harvey et al., 1996):

(5) $$q_{s}=\alpha \cdot A\cdot L$$

The hydrological retention factor (*R*_H_ [T/L]) is a useful metric to compare transient storage among reaches and under different discharges. *R*_H_ quantifies the storage zone residence time of water per unit of stream reach traveled and it can be evaluated as (Morrice et al., 1997):

(6) $$R_{H}=\frac{A_{TS}}{A\cdot v}$$

We evaluated *F*_MED_ [−] which incorporates the role of advective transport and transient storage processes (Runkel, 2002):

(7) $$F_{MED}≅\left(1-e^{\left(-L\frac{\alpha }{v}\right)}\right)\frac{A_{TS}}{A_{TS}+A}$$

*F*_MED_ is the fraction of median travel time due to transient storage (or percent if multiplied by 100). Increasing values of *F*_MED_ have to be interpreted as relatively larger importance of the transient storage processes on the solute transport in the stream corridor.

From the measured water depth in the stream channel and other streambed characteristics we evaluated the Darcy‐Weisbach friction factor (*f* [−]). This quantity has been related to streambed complexity and in‐stream storage zones and its formulation reads (Bencala & Walters, 1983; Hart et al., 1999; Thackston & Schnelle, 1970):

(8) $$f=\frac{8g\cdot d\cdot S}{v^{2}}$$

where *g* [L/T^2^] is the gravitational constant, *S* [L/L] is the slope of the energy grade line estimated from the stream channel slope (Zarnetske et al., 2007), *d* [L] is the average water depth measured in the stream channel.

We evaluated Manning's roughness coefficient *n* [−] to assess whether an increase in transient storage area is linked to an increase of friction with in‐stream sediments due to a larger contact area with the streambed fractured slate:

(9) $$n=\frac{H_{R}^{\frac{2}{3}}\cdot S^{\frac{1}{2}}}{v}$$

## 3 Results

### 3.1 Transient Storage Model Parameters and Their Identifiability

**Figure 2:**
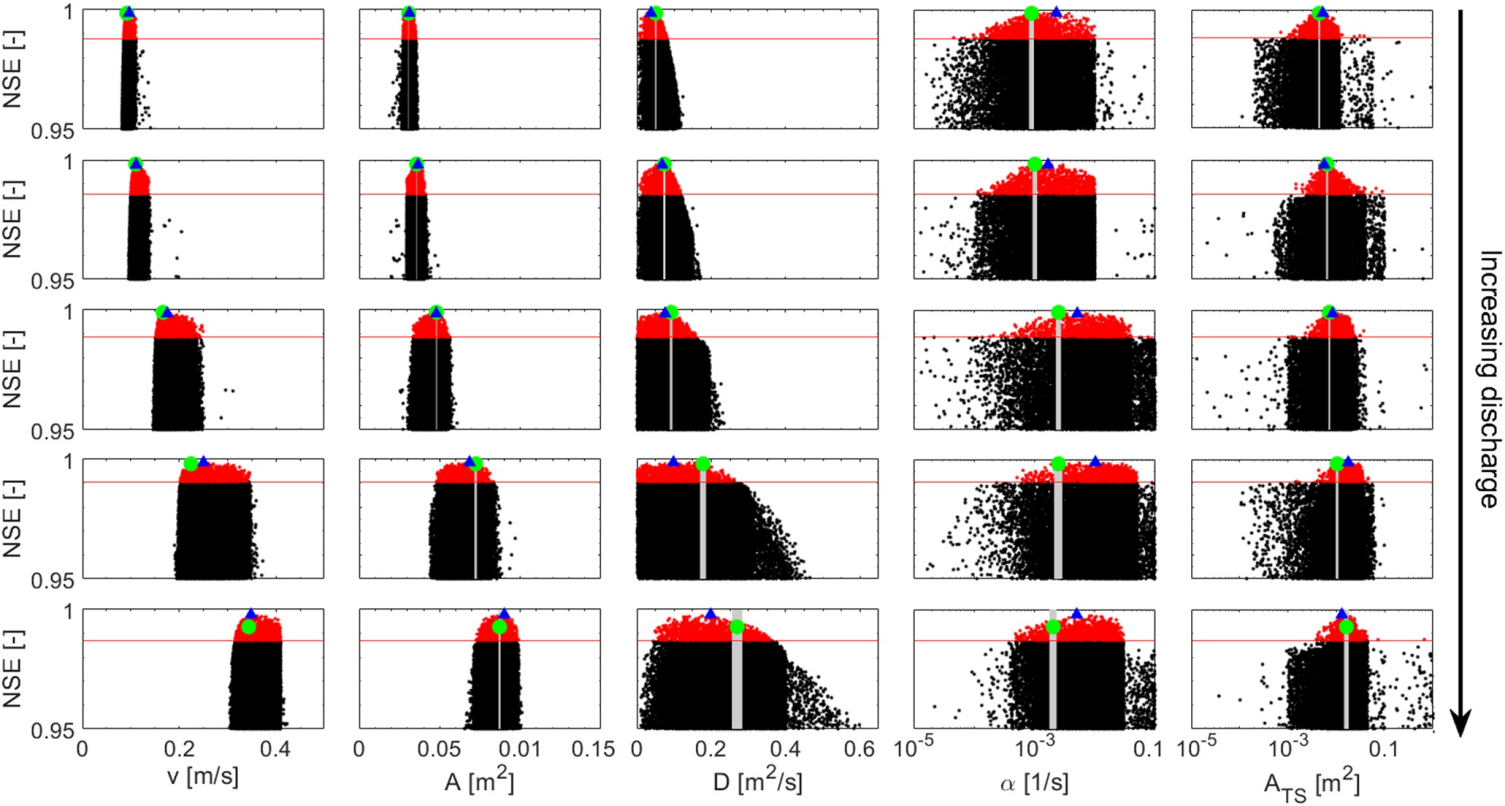
Results of Global identifiability analysis and Dynamic identifiability analysis (GlaDy) identifiability analysis reported as parameter values plotted against the corresponding NSE values. Black and red dots indicate parameter sets and corresponding model performances with worse (black) and better (red) performance than the ADE. Green dots indicate calibration results via OTIS‐P and the gray areas indicate the corresponding 95% parameter confidence limits. The blue triangles indicate the best‐performing parameter set obtained via the GlaDy identifiability analysis. The horizontal red line indicates the adopted behavioral threshold (NSE_ADE_). Results are reported for five different experiments with higher values of discharge: first row: E290 = 2.8 liters/s; Second row: E04 = 3.8 liters/s; Third row: E06 = 7.9 liters/s; fourth row: E10 = 15.9 liters/s; Fifth row: E14 = 28.6 liters/s.

The GlaDy identifiability analysis approach was effective in identifying the model parameters for the 30 tracer experiments regardless of the hydrologic conditions (Figure 2). The best‐fitting parameter sets obtained at the end of the TSM iterations outperformed in terms of objective function NSE the OTIS‐P results for 20 of the 30 experiments (Table S1 in Supporting Information S1). OTIS‐P also proved ineffective in calibrating the TSM parameters for three tracer experiments, due to convergence errors in the inverse modeling scheme. The stream discharge assessed through the GlaDy identifiability analysis exhibited values that closely aligned with those derived from the dilution gauging method. This was evident from the plot of the product of *v∙A* from the best‐fitting parameter sets obtained via the GlaDy identifiability analysis against the discharge values obtained through the dilution gauging method, which displayed a 1:1 relationship (Figure S1 in Supporting Information S1).

**Figure 3:**
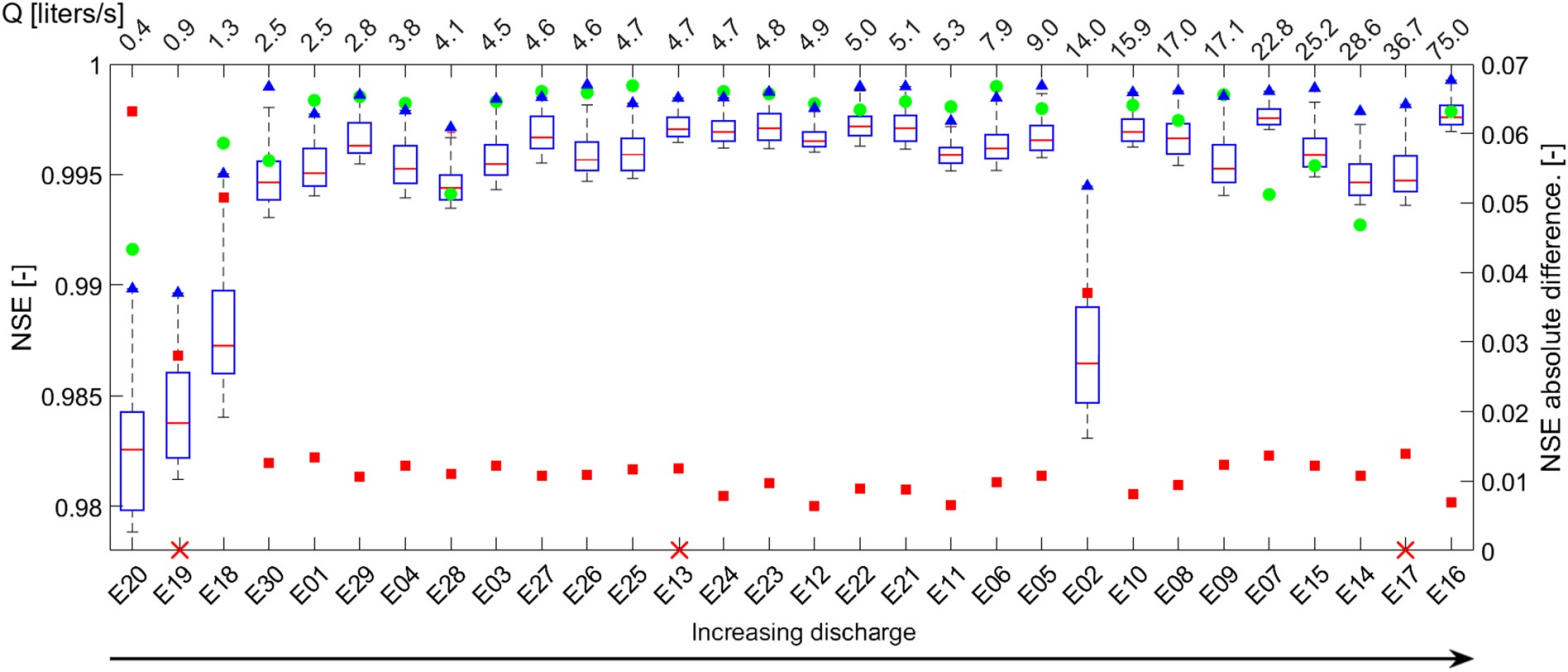
Left *y*‐axis: Boxplot of the distributions of model error for the 10% best‐performing parameter sets (NSE > NSE_ADE_). Blue triangles and green dots indicate model performances obtained via the Global identifiability analysis and Dynamic identifiability analysis (GlaDy) identifiability analysis and OTIS‐P respectively. The bottom *x*‐axis reports the ID code of the tracer experiments, while the upper *x*‐axis indicates the corresponding discharge conditions [liters/s]. Right *y*‐axis: the red squares indicate the difference between NSE_ADE_ and the NSE of the best‐performing parameter sets via the iterative modeling approach. Red crosses on the bottom *x*‐axis indicated ineffective application of OTIS‐P (false convergence).

**Figure 4:**
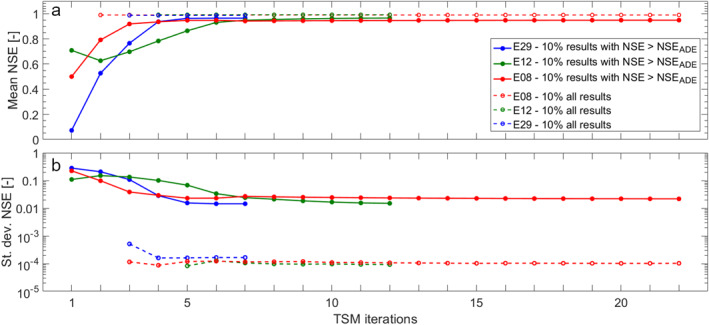
Dependency of (a) mean and (b) standard deviation of model error for the top 10% of the modeling results on the number of TSM iterations. Results have been shown for three experiments (E29 = 2.78 liters/s; E12 = 4.88 liters/s; E08 = 16.97 liters/s). Each TSM iteration includes model performances for 35,000 parameter sets. An increase in TSM iterations has to be interpreted as an increase in the total number of parameter sets considered for the evaluation of mean and standard deviation of NSE values (e.g., 10 TSM iterations include model results for 350,000 parameter sets).

The distribution of the model errors for the top 10% of model results with NSE > NSE_ADE_ indicates that higher discharge during an experiment is linked to a better performance of the TSM compared to experiments with lower discharge (boxplots, Figure 3). This is observable in the results from the iterative modeling approach and OTIS‐P (blue triangles and green dots in Figure 3) and by the fact that the Spearman correlation coefficient (*Rs*) of NSE_ADE_ for increasing discharge was positive and significant (*Rs* = 3.996, with *p*‐*value* < 0.05). The difference in performance between the TSM results and the NSE_ADE_ is smaller for experiments with higher discharge compared to experiments with low discharge (red squares, Figure 3), however this difference was not significantly correlated with discharge (*p‐*value > 0.05). Our results also show that after four or five TSM iterations the mean and standard deviation of the model errors for the top 10% of model results and for the top 10% of model results with NSE > NSE_ADE_ are constant with the increasing number of iterations (Figure 4). This outcome shows that the high number of iterations only matters for obtaining the best‐fitting parameter sets (number of red dots in Figure 2 and blue triangles in Figures 2 and 3) but does not control the distribution of the behavioral parameter sets. This is because model performances for the top 10% of model outcomes converge toward unique values after a few TSM iterations and do not decrease considerably with the number of iterations (Figure 4). The GlaDy identifiability analysis also shows that under low discharge there is a sharp decrease in model errors with the number of TSM iterations, and that the behavioral threshold of obtaining at least 1,000 parameter sets with NSE > NSE_ADE_ is satisfied after a few iterations (<10 iterations, blue lines Figure 4). On the contrary, under higher discharge, the GlaDy identifiability analysis shows a smoother decrease of model performances with the increasing number of model iterations, and the behavioral threshold of obtaining at least 1,000 parameter sets with NSE > NSE_ADE_ is satisfied only after a large number of iterations (>10 iterations, red lines Figure 4).

### 3.2 Extension and Contraction of the Hyporheic Zone and Development of In‐Stream Storage Zones

**Figure 5:**
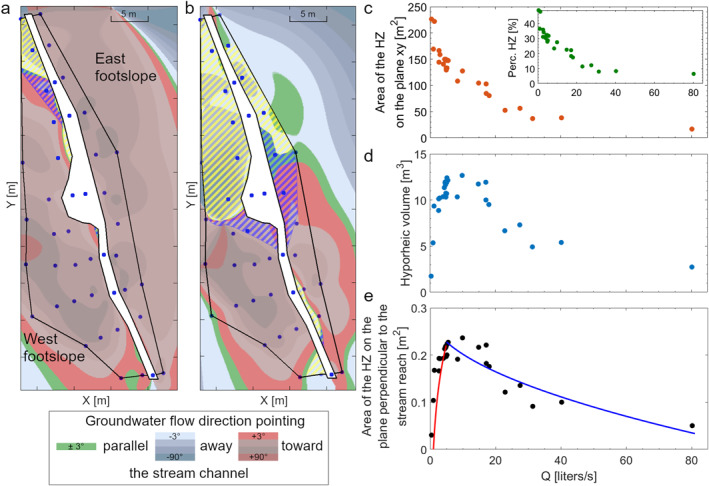
Contraction and extension of the size of the hyporheic zone exemplified for two experiments with (a) 75.0 liters/s (E16) and (b) 0.4 liters/s (E20). The blue dots in (a) and (b) indicate the location of the groundwater monitoring well network used to measure the groundwater table during each tracer injection. The colored areas in (a) and (b) indicate the groundwater flow direction normalized with the respect to the direction of the stream channel on the *xy* plane (flow direction of 0° equal to −72° on the *xy* plane). Green areas indicate groundwater flow direction flowing parallel to the stream channel; blue areas indicate groundwater pointing away from the stream channel; red areas indicate groundwater pointing toward the stream channel. The dashed yellow areas indicate the area of the hyporheic zone receiving stream water, while the dashed blue areas indicate the area of the hyporheic zone returning water to the stream channel. Together, the yellow and the blue dashed area indicate the area of the hyporheic zone on the *xy* plane. The black line indicates the perimeter of the maximum size of the hyporheic zone conditional to the well network. (c) Orange dots, dependency of the evaluated size of the hyporheic zone on the *xy* plane with discharge. The subplot (green dots) reports the same dependency, but indicated via the percentage area of the HZ over the maximum near‐stream GW area in the *xy* plane. (d) Dependency of the evaluated volume of the hyporheic zone with discharge. (e) Dependency of the area of the hyporheic zone on the plane perpendicular to the stream reach with discharge. The red and blue curves indicate the best‐fitting relationships for values below and after 5 liters/s.

To aid the interpretation of the TSM results we evaluated the size of the hyporheic zone during each tracer experiment by calculating the area of the near‐stream groundwater pointing away and then returning toward the stream channel using the near‐stream groundwater monitoring well network (cfr. Section 2.3). Groundwater table observations and the groundwater flow direction indicate that the hyporheic zone is smaller when discharge is higher (Figure 5). The size of the hyporheic zone on the *xy* plane varies between 29.79 m^2^ at 75 liters/s (E16, Figures 5a) and 226.34 m^2^ at 0.4 liters/s (E20, Figure 5b) which are 6.4% and 48.8% of the maximum size of the hyporheic zone (black perimeter, Figures 5a and 5b). The size of the hyporheic zone significantly decreases under higher discharge (Figure 5c, *Rs =* −0.90, *p‐value* < 0.01). Despite the size of the hyporheic zone on the *xy* plane decreases with discharge, the water depth in the stream channel has a logarithmic trend with the discharge (*R* ^*2*^ = 0.81, *p‐value* < 0.01, and *Rs =* 0.85, *p‐value* < 0.01, plot not shown). As a result, the volume of the hyporheic zone displays an increase with discharge for values below ∼5 liters/s and a decrease with discharge for values above ∼5 liters/s (Figure 5d). The normalization of the volume of the hyporheic zone over the reach length allowed us to evaluate the change in the average size of the area of the hyporheic zone perpendicular to the stream reach with varying discharge conditions (Figure 5e). This relationship was well approximated by a logarithmic increase (*R* ^*2*^ = 0.919, *p‐value* < 0.01, red line in Figure 5e) for discharge values lower than 5 liters/s, and by a power law (*R* ^*2*^ = 8.24, *p‐value* < 0.01, blue line in Figure 5e) for discharge values above than 5 liters/s.

**Figure 6:**
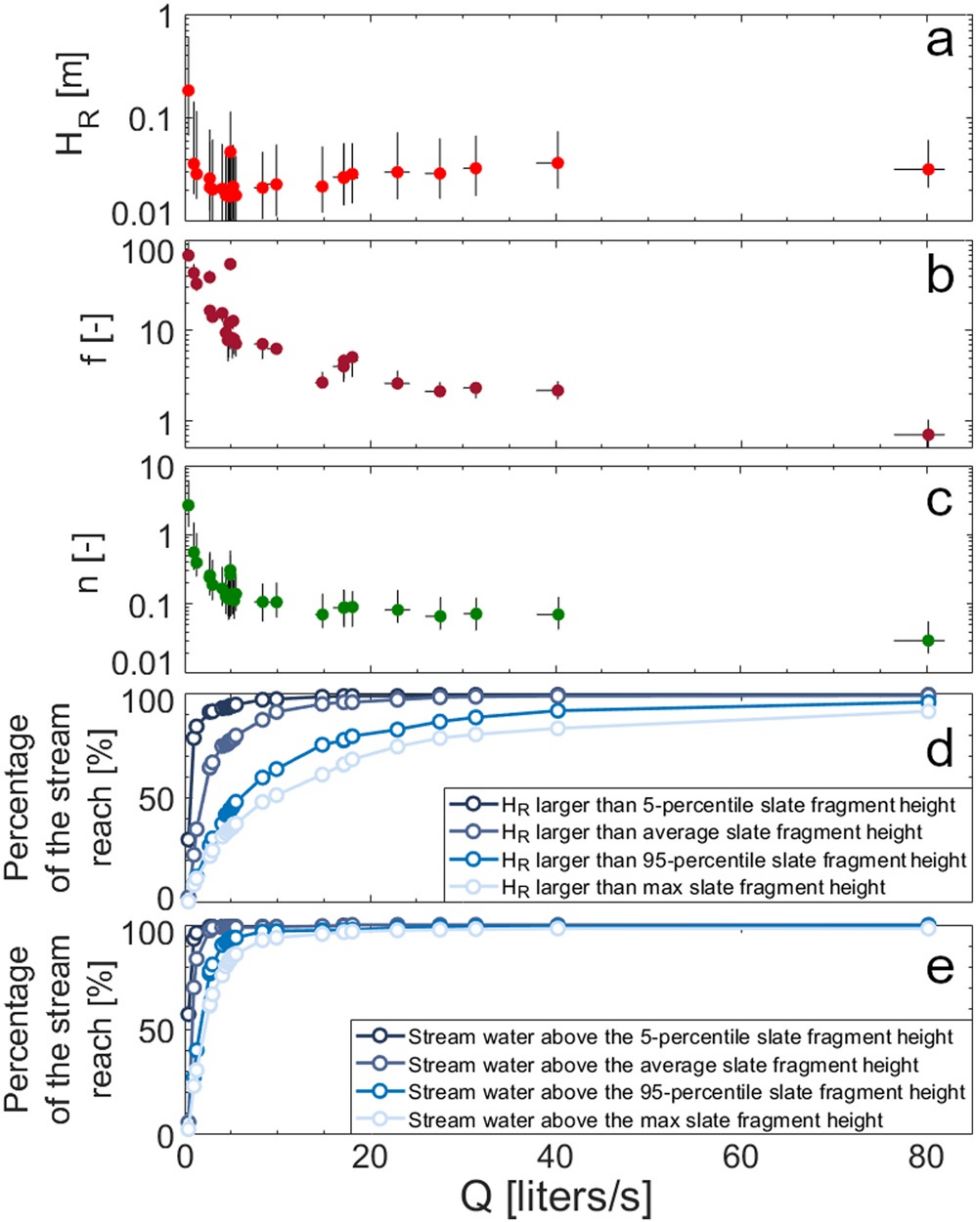
Evaluation of (a) hydraulic radius *H*_*R*_, (b) Darcy‐Weisbach friction factor *f*, (c) Manning's roughness coefficient *n*, and percentage of the stream reach where the (d) hydraulic radius or the (e) stream water level was higher than the 5‐percentile, average, 95‐percentile and maximum height of the slate fragment that makes up the streambed against discharge for the 30 tracer experiments. Horizontal black lines indicate the 5‐ and 95‐percentile limits of discharge for the top 10% TSM results with NSE > NSE_ADE_. Vertical black lines indicate the 5 and 95 percentile limits of *H*_*R*_, *f*, and *n* evaluated for the total LIDAR transects.

The hydraulic radius *H*_*R*_ decreases for discharge between 0 and 5 liters/s and increases with discharge for values above 5 liters/s (Figure 6a). The Darcy‐Weisbach friction factor *f* significantly decreases with higher discharge (Figure 6b, *Rs* = −0.885, *p‐value* < 0.01) and it can be approximated by a power law fitting function (*R* ^*2*^ = 0.706, *p‐value* < 0.01). Similarly, Manning's roughness coefficient *n* significantly decreases with higher discharge (Figure 6c, *Rs* = −0.872, *p‐value* < 0.01) and this trend is well approximated by an exponential function (*R* ^*2*^ = 0.977, *p‐value* < 0.01). The size of the slate fragments above the talweg evaluated for each LIDAR transect allows us to extrapolate how many stream transects (evaluated as a % over the total) have a water table and hydraulic radius above a certain height of the slate fragments (reported as 5‐percentile, average, 95‐percentile, and a maximum of the slate height, Figures 6d and 6e). Our results show that the hydraulic radius *H*_*R*_ is above the 5‐percentile, the average, and the 95‐percentile of the fragment height for the majority of the stream reach (95%) when discharge is above 7.9, 15.9, and 75 liters/s respectively (Figure 6d). Similarly, the measured stream water level is above the 5‐percentile, the average, the 95‐percentile, and the maximum size of the height of the fragmented slates in most of the stream reach (95%) when discharge is above 1.3, 2.5, 7.9, and 14 liters/s, respectively.

### 3.3 How Does Transient Storage Change Between Experiments?

**Figure 7:**
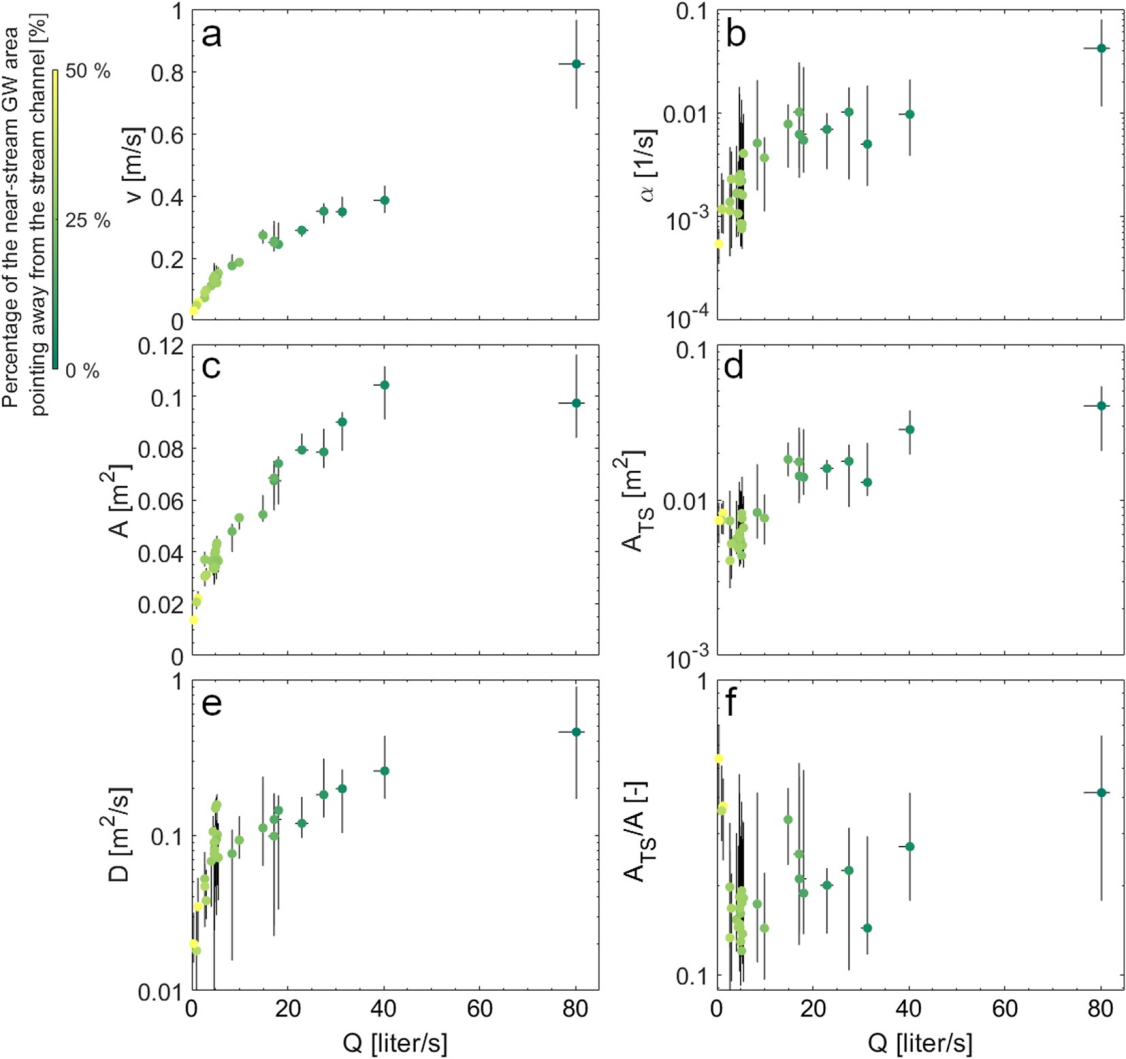
TSM parameters in relation to discharge during the experiments. Plots are reported for (a) flow velocity, (c) advective channel cross‐sectional area, (e) longitudinal dispersion coefficient, (b) transient storage exchange rate, (d) transient storage cross‐sectional area, and (f) ratio between transient storage cross‐sectional area and advective channel cross‐sectional area. Dots show the best parameter set obtained from the iterative modeling approach. Vertical and horizontal black lines indicate 5 and 95 percentile limits of the top 10% results with NSE > NSE_ADE_. Gradient colors indicate the percentage of the near‐stream groundwater receiving and returning stream water to the stream channel (cfr. Figure 5).

Our results from the GlaDy identifiability analysis show that advection‐dispersion parameters increase with discharge (Figures 7a, 7c and 7e). The increase of *v* with discharge is significant (*Rs* = 0.945, *p‐value* < 0.01) and follows a linear and power law function (*R* ^*2*^ = 0.971, *R* ^*2*^ = 0.975, respectively; *p‐value* < 0.01). The increase of *A* and *D* with discharge is significant (*Rs* = 0.932 and *Rs* = 0.833, respectively, with *p‐value* < 0.01) following a power law increase (*R* ^*2*^ = 0.937 and *R* ^*2*^ = 0.862, for *A* and *D* respectively; *p‐value* < 0.01). The TSM parameters also increase with discharge. *α* increases significantly with discharge (*Rs* = 0.784, *p‐value* < 0.01, Figure 7b) following a quadratic fitting function (*R* ^*2*^ = 0.936, *p‐value* < 0.01). *A*_TS_ shows high variability for discharge stages lower than 5 liters/s having a non‐significant correlation with discharge (*Rs* = −0.437, *p‐value* > 0.1). However, for discharge values larger than 5 liters/s the *A*_TS_ parameter significantly increases with discharge (*Rs* = 0.815, *p‐value* < 0.01) following a power law fitting function (*R* ^*2*^ = 0.895, *p‐value* < 0.01). The correlation of *A*_TS_ with discharge results in a similar behavior of *A*_TS_ */A* (Figure 7f). The ratio *A*_TS_ */A* shows a sharp decrease with discharge for values lower than ∼5 liters/s (power law *R* ^*2*^ = 0.915, *p‐value* < 0.01, with *Rs* = −0.657, *p‐value* = 0.012), and increases with discharge for values above 5 liters/s (*R* ^*2*^ = 0.566, *p‐value* < 0.01 using a power law function with *Rs* = 0.588, *p‐value* = 0.018).

**Figure 8:**
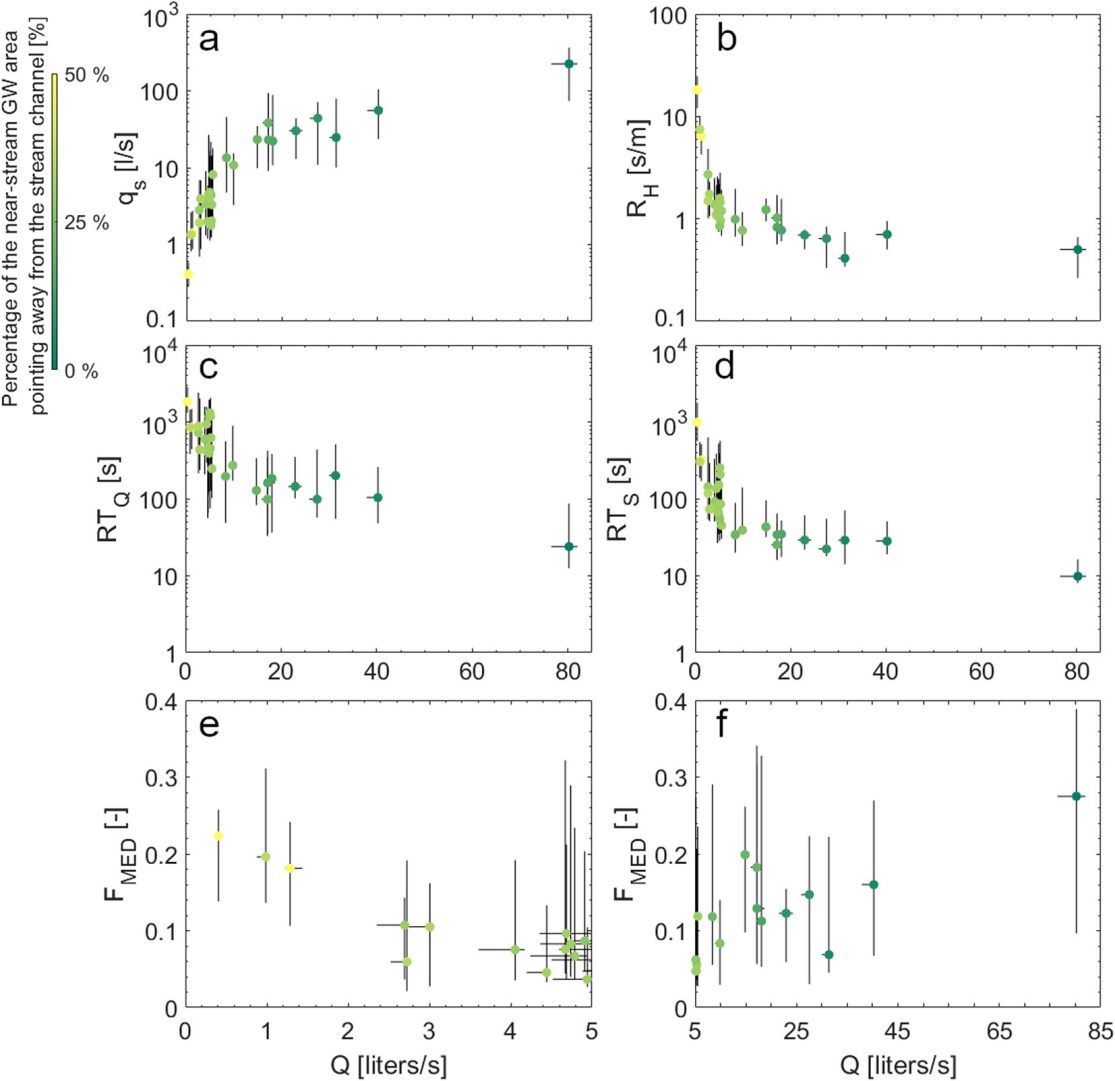
Same as Figure 7, but reporting (a) the total water flux exchanged between the stream channel and the storage zone, (b) the hydrological retention factor, (c) the average residence time of a tracer molecule in the stream channel, and (d) in the transient storage zone transport metrics as a function of discharge. The last two plots report the fraction of median travel time due to transient storage respectively for discharge conditions (e) before and (f) after 5 liters/s.

The transport metrics show different patterns against discharge (Figure 8). The total water flux exchanged between the stream channel and the storage zone (*q*_*s*_) significantly increases with discharge (*Rs* = 0.866, *p‐value* < 0.01) and this relationship was well approximated by both a power law and a cubic function (*R* ^*2*^ > 0.87, *p‐value* < 0.01, Figure 8a). The hydrologic retention factor (*R*_H_) is significant and negatively correlated with increasing discharge (*Rs* = −0.872, *p‐value* < 0.01, Figure 8b) following an exponential or a power law decrease function (*R* ^*2*^ > 0.96, *p‐value* < 0.001). The average residence time in the stream channel (*RT*_Q_) and the transient storage zone (*R* *T*_s_) are negatively and significantly correlated with discharge (*Rs* = −0.784 and *Rs* = −0.857, *p‐value* < 0.01, Figures 8c and 8d respectively). The decrease of *RT*_Q_ and *R* *T*_s_ with *Q* can be well approximated by a power law (*R* ^*2*^ = 0.627 and *R* ^*2*^ = 0.932 respectively, *p‐value* < 0.01). The fraction of median travel time due to transient storage shows a decreasing and an increasing trend with increasing discharge, for values respectively below and above 5 liters/s (Figure 8 e, f) When the discharge is comprised between 0 and 5 liters/s, *F*_MED_ decreases significantly with discharge (*Rs* = −0.684, *p‐value* < 0.01) following a linear (*R* ^*2*^ = 0.776, *p‐value* < 0.01) or a power law (0.851, *p‐value* < 0.01) decrease. However, when the discharge is above 5 liters/s, *F*_MED_ increases significantly with discharge (*Rs* = 0.691, *p‐value* < 0.01) following a linear (*R* ^*2*^ = 0.558, *p‐value* < 0.01) or a power law (*R* ^*2*^ = 0.567, *p‐value* < 0.01) increase.

## 4 Discussion

### 4.1 Parameter Identifiability in the Transient Storage Model Depends on Discharge During the Tracer Experiments

When TSM parameters are non‐identifiable they are interdependent and a change of a certain parameter would be balanced by a proportional change of other parameters, eventually leading to the same model performances (Camacho & González, 2008; Kelleher et al., 2019; Wagener, Lees, & Wheater, 2002; Wlostowski et al., 2013). Compared to the available literature, no previous study directly addressed the role of increasing discharge on the identifiability of TSM parameters. Our results indicate that the interdependency of TSM parameters increases with higher discharge during the experiment. This is visible from the less pronounced peak of performances of *v*, *A,* and *D* (red dots, Figure 2) and the fact that the space of the advection‐dispersion parameters showing satisfactory model performances (NSE > NSE_ADE_) increases with the discharge (red dots, Figure 2). This results in a larger 5‐ and 95‐percentile limits of the top 10% TSM results compared to experiments with low discharge, where advection‐dispersion parameters show a rather narrow peak of performances (Figure 2, Table S1 in Supporting Information S1; black lines, Figure 7).

Our outcomes indicate that the advection‐dispersion parameters explain largely the shape of BTC during high discharge and that the transient storage process added to the advection‐dispersion equation contributes to a modest improvement of model performances compared to the ADE. Since larger portions of the BTC can be explained by the advection‐dispersion process it may be argued that the transient storage parameters are less identifiable because the transient storage process is less important with increasing discharge. This interpretation is corroborated by the obtained significant correlation between NSE_ADE_ with increasing discharge and the limited increase of the TSM performances compared to the ADE performances with higher discharge (red squares, Figure 3).

The observed parameter interaction together with the larger number of TSM iterations and sampled parameter sets needed to obtain identifiable TSM parameters at higher discharge conditions (Figure 4) support the interpretation that greater parameter interactions cause poorer parameter identifiability and model performances (Kelleher et al., 2013; Ward, Payn, et al., 2013). This study positions itself alongside other studies that investigate the identifiability of TSM parameters and show that the advection‐dispersion process becomes predominant over the transient storage processes during higher discharge. For example, Wagner and Harvey (1997) were the first to point toward the role of the advection‐dispersion process for the identifiability of the transient storage parameters while Kelleher et al. (2013) and Bonanno, Blöschl, and Klaus (2022) demonstrated advection‐dispersion parameters control progressively larger portions of the BTC under higher discharge, while transient‐storage parameters control progressively larger portions of the BTC under lower discharge.

The results reported in this study present a novel viewpoint and a potential elucidation for the non‐identifiability reported in prior investigations, where the efficacy of employing random sampling to estimate TSM parameters has been inconsistent, with no clear understanding of the underlying reasons (see Table 1). We believe that the advection‐dispersion process was not predominant at study sites with relatively low discharge meaning that *α* and *A*_TS_ were explanatory of large portions of the BTC. As a result, the TSM improved substantially performances compared to ADE due to the pronounced tail of the BTC, and the identifiability of TSM parameters was achieved via two iterative random sampling for a total of 100,000 parameter sets (Ward et al., 2018; first BTC in Ward, Kelleher, et al., 2017). On the other hand, headwater reaches characterized by steep channel gradients and relatively short investigated reaches indicated non‐identifiability of TSM parameters (Kelleher et al., 2013; Ward, Payn, et al., 2013; second and third BTCs in Ward, Kelleher, et al., 2017). This is probably because the advection‐dispersion process at these study sites dominated the tracer transport and the investigated parameter space and/or the used number of parameter sets (often ≤100,000) did not allow to target NSE > NSE_ADE_ for a sufficient number of parameter sets to show identifiability.

The GlaDy identifiability analysis introduced in Bonanno, Blöschl, and Klaus (2022) and used in this work can also demonstrate that selecting a narrow (< two orders of magnitude) parameter interval in a random‐sampling approach can cause an “apparent” non‐identifiability in TSM. If we had sampled a parameter from a narrow space around the peak of performance (e.g., *α* between 0.001 and 0.003 s^−1^ for experiment E06, results not shown), the identifiability analysis results would lead us to the conclusion that the parameter was non‐identifiable. However, this same interval shows optimal performances when a wider parameter space is sampled (Figure 2). This may account for the lack of parameter identifiability observed in previous studies that have focused on a narrow range of TSM parameters (Camacho & González, 2008; Wagener, Lees, & Wheater, 2002; Wlostowski et al., 2013), as a limited parameter space may not permit a clear improvement in model performance. Consequently, the identifiability of TSM parameters could remain hidden, especially if an insufficient number of parameter sets are sampled (<100,000, Bonanno, Blöschl, & Klaus, 2022; Ward et al., 2017). Our findings demonstrate that the GlaDy identifiability analysis can address parameter identifiability across a parameter space spanning several orders of magnitude, which is consistent with recent recommendations for identifiability analysis (Pianosi et al., 2016) and prior research (Kelleher et al., 2013; Kelleher et al., 2019; Ward, Kelleher, et al., 2017; Ward, Payn, et al., 2013).

Our OTIS‐P simulations demonstrated satisfactory model performance, as the calibrated TSM parameters were within the same parameter space as those obtained from the GlaDy identifiability analysis (Figure 2; SI). While these outcomes suggest that OTIS‐P can yield robust results, past work highlighted that OTIS‐P does not provide information about the identifiability and performances of TSM parameters across their feasible range, making it challenging to determine if OTIS‐P results shall be used for process interpretation or not (Kelleher et al., 2013; Knapp & Kelleher, 2020; Ward, Kelleher, et al., 2017). Moreover, the best‐fitting parameter sets obtained from the GlaDy identifiability analysis outperformed in terms of objective function (NSE) the OTIS‐P results for most tracer experiments and allowed us to obtain parameter identifiability even for BTCs where OTIS‐P failed to converge (Table S1 in Supporting Information S1). Furthermore, it is important to note that while GlaDy identifiability analysis demonstrated superior performance compared to OTIS‐P for 20 of the 30 experiments, the difference in terms of NSE objective function was negligible for many of them (Table S1 in Supporting Information S1). Therefore, for future studies investigating TSM it may be advisable to use random sampling over a parameter space defined as a neighborhood range of TSM parameters obtained via OTIS‐P. However, in cases where OTIS‐P fails to converge or when TSM parameters cannot be identified through classic random‐sampling approaches, the GlaDy identifiability analysis has been demonstrated to be a powerful and flexible tool for achieving systematic parameter identifiability across a wide range of hydrologic conditions. This represents a significant and previously unreported achievement in TSMs.

The performances of OTIS‐P and the GlaDy identifiability analysis reported in this study are certainly typical of the study site. Future research is necessary to evaluate the applicability of the GlaDy identifiability analysis to other stream reaches, as tracer experiments conducted in different geomorphological settings have been shown to strongly influence the shape of the BTC and TSM performances (D’Angelo et al., 1993; Edwardson et al., 2003; Hall et al., 2002; Zarnetske et al., 2007). Additionally, the ability to obtain parameter identifiability may have been facilitated by the simplistic formulation of the TSM, which treats transient storage as a single storage area with a solute residence time that follows an exponential decay. Future work should test the GlaDy identifiability analysis with modifications to TSM that increase the number of parameters, thus their interaction and the potential for non‐identifiability (Knapp & Kelleher, 2020). Among the diverse TSM formulations aimed at providing a more realistic representation of solute transport in streams, the GlaDy identifiability analysis should be applied to TSMs with multiple transient storage areas (Choi et al., 2000; Fabian et al., 2011), TSMs with different residence time distribution laws (Bottacin‐Busolin & Marion, 2010; Marion et al., 2008; Gooseff et al., 2005; Haggerty et al., 2002), and BTCs of non‐conservative solutes (Kelleher et al., 2019).

### 4.2 Dynamics of Transient Storage Processes Under Different Hydrologic Conditions

The higher longitudinal dispersion coefficients *D* in experiments with higher discharge are also in line with the TSM formulation and with the observed increase of the Reynolds number with discharge (between 2.99·10^3^ and 2.22·10^5^, linear and quadratic function fit with *R* ^*2*^ > 0.99, *p‐value* < 0.01, plots not shown). *D* is responsible for the longitudinal spreading of the tracer above and behind the center of the solute pulse, thus it is expected to increase with increasing streambed roughness and channel complexity (Gooseff, Bencala, et al., 2008). Furthermore, *D* is proportional to the spatial variability of the flow velocity across the velocity field, thus it increases with discharge as a result of increases in wetted stream area and the consequent spatial heterogeneity in the velocity profile. This is despite increasing turbulence with discharge would theoretically slightly decrease *D* (Fischer et al., 1979). Similarly, many experimental studies report that *D* is proportional to discharge (Kashefipour & Falconer, 2002).

The trend of *F*_MED_ for discharge below 5 liters/s (Figure 8e) together with the large size of the hyporheic area and volume assessed through groundwater measurements (Figures 5c–5e) suggest a non‐negligible contribution of hyporheic exchange to transient storage for experiments with low discharge (<5 liters/s) compared to experiments with higher discharge. This interpretation is also supported by the *A*_TS_ */A* ratio for discharge below 5 liters/s (Figure 7f), the high values of the hydrologic retention factor *R*_H_ (>1 s/m Figure 8b), and the negative correlation of *RT*_s_ and *RT*_Q_ with discharge (Figures 8c and 8d).

**Figure 9:**
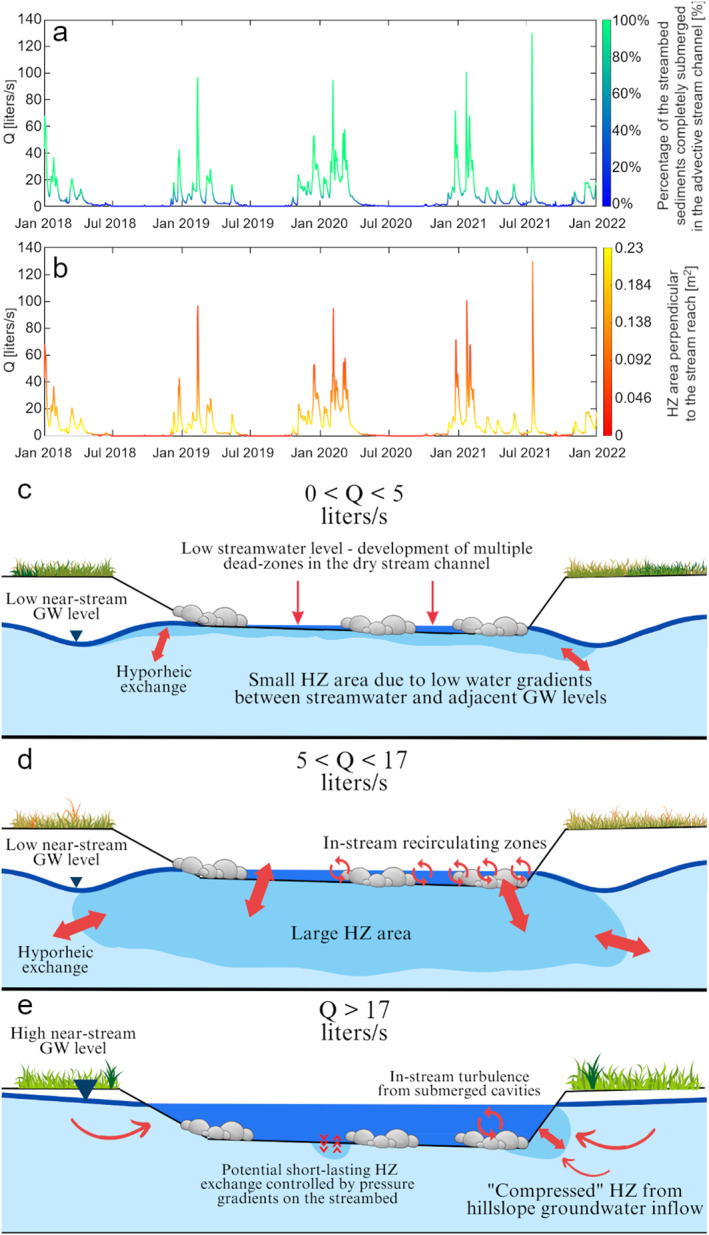
Hydrograph of the Weierbach catchment for four hydrologic years considering (a) the increase of the streambed sediments completely submerged in the advective stream channel and (b) the increase and decrease of the hyporheic zone area perpendicular to the stream channel. (c–e) Figure reports a perceptual model of the mechanisms controlling the transient storage in the investigated stream reach with increasing discharge conditions.

This result is consistent with previous investigations at the study sites reporting low near‐stream groundwater table as a result of a discontinued hillslope‐riparian‐stream connectivity during dry hydrologic conditions (Bonanno et al., 2021; Figure 9a). Our TSM results are consistent with previous studies where gradients from the stream channel toward the adjacent groundwater have been linked to hyporheic transport (González‐Pinzón et al., 2015; Harvey & Bencala, 1993; Kasahara & Wondzell, 2003). However, our findings also indicate a significant role of in‐stream transient storage during low discharge (<5 liters/s). This can be deduced from the high values of the friction factor *f* and the roughness coefficient *n* obtained for experiments with low discharge and the fact that the hydraulic radius *H*_*R*_ was at its minimum at *Q ∼* 5 liters/s. These results indicate that the wetted perimeter increases more than the wet stream area for higher discharge between 0 and ∼5 liters/s. This in turn causes a greater proportion of the streambed material to be submerged, but not completely (Figures 6d and 6e). This partial submergence of larger areas of the streambed material causes the development of secondary flowpaths among the slate fragments and turbulences in the shaded area immediately downstream causing an increase in the in‐stream transient storage. Our results show that transient storage during experiments under low discharge at the study site (*Q* < 5 liters/s) cannot be explained by hyporheic exchange or in‐stream transient storage alone, but as a combination of both (Figure 9c).

Higher discharge at the study site is characterized by an increase in gradients from the adjacent groundwater toward the stream channel, indicating a persistent hillslope‐stream connectivity on the west and the east hillslopes (Bonanno et al., 2021). This is consistent with our results indicating a decrease in the size of the groundwater area and volume receiving water from the stream channel (Figures 4c and 4d) for experiments with discharge higher than 5 liters/s. These results show that the hyporheic exchange decreases with higher discharge suggesting that transient storage is mainly controlled by in‐stream transient storage. However, the observed high percentage of slate fragments in the stream channel that are entirely submerged below the water table with higher discharge (Figures 6d and 6e) indicates that the secondary flowpaths and recirculation zones controlling in‐stream transient storage at lower discharge are now part of the advective stream channel (Figure 9e). Also, the observed trend of the hydraulic radius with discharge shows that the wet area increases more than the wet perimeter for discharge higher than 5 liters/s (Figures 6a–6c). These results provide additional evidence that also in‐stream transient storage becomes less important for solute transport with higher discharge at the study site.

As stream discharge increases further, the streambed sediments become completely submerged below the in‐stream water level (*Q* > 17 liters/s, Figure 6e) and the hyporheic zone area and volume progressively decrease (Figures 5c–5e). Also, the decrease of the roughness *n* and friction factor *f* (Figures 6b and 6c) indicates a decreasing shear velocity u* with increasing discharge. This result suggests a lower contribution of shear stress to in‐stream transient storage controlled by turbulences (u* ∝ gdS as in Equation 8, see Fischer et al., 1979). Instead, transient storage at the study site might be ruled by increasing spatial heterogeneities of the velocity gradients due to the increasing wetted area with higher discharge, by the occurrence of recirculating zones above submerged cavities and slates in the streambed (Jackson et al., 2013), or by short‐lasting hyporheic exchange controlled by local pressure gradients of the stream water on the streambed (Cardenas & Wilson, 2007). This interpretation aligns with the trend observed in the evaluated *F*_MED_ and *RT*_s_ metrics, which indicate that transient storage becomes increasingly relevant with higher discharge, but lasting only a few seconds within the stream corridor (Figures 8d and 8f). This outcome is consistent with research at other sites characterized by low hyporheic exchange, where higher discharge during tracer experiments resulted in relatively lower in‐stream transient storage compared to lower discharge (Martí et al., 1997; Zarnetske et al., 2011). However, our results on *F*_MED_ demonstrate that an increase in discharge does not necessarily indicate an absence of in‐stream storage (Figure 8f). Instead, our outcomes suggest that the underlying mechanisms controlling in‐stream storage change with increasing discharge and shift from secondary flowpaths and recirculating zones due to partially submerged streambed materials (Figures 5e and 9d) to increasing velocity gradients in larger wetted areas and recirculating eddies in the water column probably due to submerged streambed cavities (Gooseff, Payn, et al., 2008; Jackson et al., 2013).

Compared to the extension of the hyporheic zone area and volume (Figures 4c and 4d), the deduced order of magnitude of *A*_TS_ and *q*_*S*_ (Figures 7d and 8a) indicates that we were likely unable to capture longer flowpaths and residence time of the stream water into large areas of the hyporheic zone as evaluated via the groundwater measurements. This is particularly evident when the area of the hyporheic zone perpendicular to the stream channel (Figure 5e) is compared to the TSM parameter *A*_TS_ (Figure 7d). Our results show that *A*_TS_ underestimates the area of the hyporheic zone at low discharge conditions and especially around 5 liters/s. However, with increasing discharge the *A*_TS_ parameter increases and approaches the value of the hyporheic zone area experimentally measured and averaged on the reach length (Figure 5e). This result highlights the limitations of using tracer experiments alone to capture the magnitude of the hyporheic zone and exchange, particularly at lower discharge conditions. This is because while previous studies reported instantaneous injections to be capable of returning model information comparable to that of continuous injections for conservative tracers (Gooseff, Payn, et al., 2008; Payn et al., 2008), other studies also highlight instantaneous injections are limited by the available “window of detection”, which is biased toward faster transient storage processes and shallow hyporheic exchange (Harvey & Wagner, 2000; Jin & Ward, 2005; Wondzell, 2006).

Our study is the first study to our knowledge that is addressing the concurrence of different processes controlling the transient storage via the use of TSM parameters, the near‐stream groundwater levels, and the streambed micro‐topography under several hydrologic conditions. We recognize that the adopted strategy is not without criticism. As an example, the groundwater monitoring well network is not designed to capture pressure gradients at the surface water–streambed interface that is recognized to be a non‐negligible source of hyporheic flowpaths with increasing turbulence and discharge (Cardenas & Wilson, 2007; Packman & Bencala, 2000). Also, the evaluation of the hyporheic zone on the *xy* plane is limited by the available number of wells at the study site, meaning that it could potentially be larger than the one we estimated, especially at low discharge conditions (Figures 5a and 5c). In addition, the LIDAR scans might not be representative of the streambed micro‐topography and slate distribution above the talweg across the relatively long investigated period (from December 2018 to June 2021) and more scans could have provided more robust results. Despite some limitations, our approach bypassed the “window of detection” problem typical of tracer injections and assessed the dynamic role of hyporheic exchange and in‐stream transient storage on water transport across the hydrologic year.

### 4.3 A Dynamic Perceptual Model of Transient Storage Processes

We acknowledge that our TSM results and interpretation are likely representatives of the studied reach only. However, our findings underscore the importance of investigating a wide range of hydrologic conditions to establish a robust understanding of the processes controlling transient storage in stream corridors. We used the obtained relationships between increasing discharge with the transient storage area perpendicular to the stream channel (Figure 5e) and the amount of streambed sediments completely submerged in the advective stream channel (Figure 6e) to derive the potential role of hyporheic extension and in‐stream transient storage across 4 years in the Weierbach catchment (Figures 9a and 9b). Due to the obtained relationships between TSM parameters and discharge, these plots are indicative of the expected role of in‐stream and hyporheic storage processes occurring at the study sites for a large spectrum of hydrologic conditions and in absence of tracer experiments. The results collected in this work also allowed us to build a perceptual model for a qualitative representation of the mechanisms controlling the transient storage at the study site under varying discharge (Figures 9c–9e). This is a step forward in one of the current research frontiers in hydrology, namely the spatial and temporal development of the interfaces between the stream water and the adjacent groundwater (Blöschl et al., 2019), which was argued to be caused by limited experimental measurements in the stream corridor (Ward & Packman, 2019), parameters non‐identifiability (Knapp & Kelleher, 2020), and the low amount of investigated hydrologic conditions (Ward, 2016).

Under low discharge conditions (*Q* < 5 liters/s) the extension of the hyporheic zone on the *xy* plane is large (Figures 5b and 5c). However, the low water level in the stream channel does not allow the development of a relevant gradient between the in‐stream water level and the adjacent groundwater, resulting in a small hyporheic volume and area of hyporheic zone perpendicular to the stream reach (Figures 5d and 5, 9). Also, the majority of the streambed sediments are only partially submerged (Figures 6d and 6e an 9a), which allows the development of multiple flowpaths in the stream channel in an almost‐dry streambed (Figure 9a). These mechanisms have a relatively high impact on transient storage, as also indicated by the increasing *F*_MED_ and the trend of the other transport metrics (Figure 8). With the progressive increase of discharge, the wet perimeter increases more than the wetted area until ∼5 liters/s (Figure 6a) and many in‐stream sediments get submerged, but not completely (Figures 6d and 6e and 9a). At this stage, the groundwater level adjacent to the stream channel is low enough to allow a large portion of the stream reach to be in losing condition toward the groundwater (Figures 5b and 5c), while the increasing water level in the stream channel control the development of increasing volumes of hyporheic zones (Figures 5d and 5e). These hydrologic conditions create a stage where there is a large potential for in‐stream recirculating zones and hyporheic exchange, but where the overall transient storage effect obtained by TSM is lower compared to lower hydrologic conditions (Figures 7, 8, and 9d), probably as a result of the “window of detection” problem typical of instantaneous tracer injections. With increasing wetness conditions the wet area of the stream channel increases more than the wet perimeter (Figure 6a) and in‐stream sediments get completely submerged in the stream reach at ∼17 liters/s (Figures 6e and 9a). At this stage, the inflow from the adjacent hillslopes causes the predominance of near‐stream groundwater gradients pointing toward the stream allowing only an occasional development of hyporheic exchange (Figure 5a) and a decreasing volume and area of hyporheic zone perpendicular to the stream channel (Figures 5c–5e). As a result, transient storage might be controlled by increasing velocity gradients in larger wetted areas, recirculating zones due to submerged cavities (Jackson et al., 2013) and by short‐lasting hyporheic exchange controlled by pressure gradients that the monitoring well network at the study site is unable to capture (Cardenas & Wilson, 2007; Packman & Bencala, 2000).

This perceptual model obtained and the results reported in this work can help to understand why increasing discharge in past studies was related both to an increase and a decrease of *α* and *A*_TS_ parameters and apparent contradictory model interpretation (Table 1). Our study highlights the coexistence of many simultaneous mechanisms responsible for transient storage processes and that they can both increase and decrease with increasing discharge as a result of the complex interaction between in‐stream water level, groundwater level, in‐stream sediments, and recirculating zones. As a result, depending on a site‐specific threshold for stream water level above and below streambed material, the transient storage parameters might have shown both an increase or a decrease with increasing discharge (D’Angelo et al., 1993; Edwardson et al., 2003; Fabian et al., 2011; Martí et al., 1997; Morrice et al., 1997), similar to our outcomes for discharge values below or above 5 liters/s. TSM results in past literature have also been linked to the occurrence of hyporheic exchange, and the observed trend of transient storage parameters with the streamflow discharge has been linked to a larger or lower importance of the hyporheic exchange in the stream corridor (Gooseff et al., 2003; Schmid et al., 2010; Valett et al., 1996; Zarnetske et al., 2007). In our study, we proved that increasing transient storage parameters with discharge were both linked to a decrease and a decrease in the hyporheic exchange, highlighting the pivotal role of having near‐stream groundwater measurement for a correct interpretation of TSM parameters. Eventually, we recognize that the spatial and temporal scale of the processes investigated at our study site is not representative of transient storage processes occurring in a larger‐order stream and alluvial sites where streambed sediments are probably always completely submerged and other mechanisms could be predominant, such as meandering exchange (Boano et al., 2006), the occurrence of bars and dunes (Stonedahl et al., 2013), fine‐sediment accumulation (Ward et al., 2018), and transient storage in large ponds in the stream channel (Hall et al., 2002; Magliozzi et al., 2018; Morrice et al., 1997).

We acknowledge that obtaining high‐resolution data such as spatially dense groundwater monitoring and LIDAR scans may not be feasible for many research sites. However, our findings highlight the potential pitfalls of relying solely on phenomenological models, such as TSM, for interpreting transient storage processes. To ensure accurate characterization of hyporheic exchange, future studies should include measurements of water table gradients between the stream water and adjacent groundwater, which can be obtained through the use of relatively inexpensive shallow piezometers (Fabian et al., 2011; Nowinski et al., 2012). This would provide critical insight into whether a hyporheic exchange is occurring or if it is merely speculative. Additionally, although dry streambed microtopography may not always be available, manual sampling of in‐stream streambed sediments and comparison of their size with in‐stream water levels during tracer injections can still be valuable information for determining whether all streambeds have the potential to develop secondary flow paths and in‐stream recirculating zones, or whether they are completely submerged within the advective stream channel.

## 5 Conclusion

Answering how and why transient storage processes change with different hydrologic conditions can bring a comprehensive assessment of their spatial and temporal role in regulating water quality in stream networks. In this study, we used an iterative modeling approach (the GlaDy identifiability analysis) to obtain identifiable TSM parameters for 30 tracer breakthrough experiments at different discharges in a headwater stream reach. We combined the model results, groundwater table observations, and measurements of streambed micro‐topography to support the interpretation of different processes concurring to the transient storage of stream water through a wide range of hydrological conditions. Our work showed that the parameter space where advection‐dispersion parameters were identifiable was wider under higher discharge, thus increasing the parameter interaction in the TSM. Our outcomes can thus explain the lack of parameter identifiability in several previous TSM studies and open up new challenges to address parameter identifiability in other model formulations implemented with many transient storage areas or with different residence time distributions. The introduction of the streambed micro‐topography and groundwater table measurement provided valuable data for interpreting the TSM parameters and the transport metrics describing solute transport at the study site. Our model results showed that hyporheic exchange and in‐stream transient storage control the transient storage of stream water during low discharge. Under higher discharge, the hyporheic zone becomes progressively less important in controlling transient storage, which is rather driven by in‐stream storage probably controlled by increasing velocity gradients, pressure gradients on the streambed interface, and recirculating zones by submerged cavities. Our findings inspired the development of a perceptual model for a qualitative understanding of the processes governing transient storage at the study site. The relationships between submerged sediments and the hyporheic zone development with increasing discharge were also utilized to generate a hydrograph for the study site indicating the dynamic development of in‐stream and hyporheic storage over a 4‐year period. This outcome provides insights into the expected influence of transient storage processes prior to tracer experiments. Future work should combine tracer injections with information on streambed micro‐topography and groundwater measurements in stream reaches characterized by different morphologies and hydrologic regimes. The combination of derived patterns between discharge, hyporheic area, stream water elevation, and streambed microtopography in several study sites would be key for understanding the spatial and temporal variation of transient storage processes in stream networks and across scales.

## Supporting information

Supporting Information S1

## Data Availability

The GlaDy identifiability analysis has been introduced by Bonanno, Blöschl, and Klaus (2022) and it is available in Bonanno (2022). Data associated with this manuscript are available in Bonanno, Barnich, et al. (2022).
