# Supplementary material for: Discharge, Groundwater Gradients, and Streambed Micro‐Topography Control the Temporal Dynamics of Transient Storage in a Headwater Reach
Source: Water Resour Res. 2023 Jul 10;59(7):e2022WR034053. doi: 10.1029/2022WR034053 (PMC10909551; doi:10.1029/2022WR034053)
Supplement: Supplementary file 1 — Supporting Information S1 [file WRCR-59-e2022WR034053-s001.pdf]

# Discharge, groundwater gradients, and streambed micro-topography control the temporal dynamics of transient storage in a headwater reach

**Enrico Bonanno[1,2], Günter Blöschl[2], Julian Klaus[3]**

[1] Catchment and Eco-Hydrology Group, Luxembourg Institute of Science and Technology, Belvaux, Luxembourg.

[2] Institute of Hydraulic and Water Resources Engineering, Vienna University of Technology, Vienna, Austria.

[3] Institute of Geography, University of Bonn, Bonn, Germany.

## Supporting information (SI)

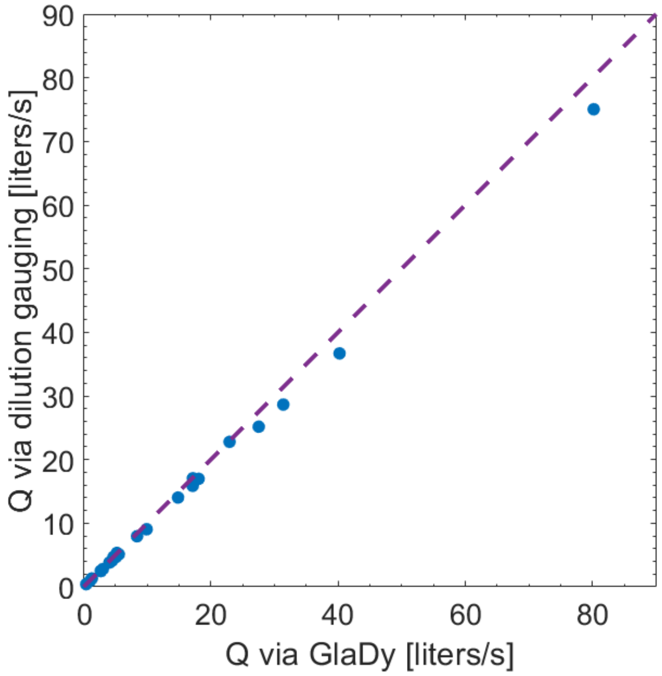


**Figure SI. Comparison of stream discharge evaluated as product of *v·A* from the best-fitting parameter sets obtained via the GlaDy identifiability analysis against the discharge values obtained through the dilution gauging method.**

**Table SI - List of the best-performing parameter sets and relative objective function performances derived via the GlaDy identifiability analysis and OTIS-P modelling approach. The best results are indicated in bold font.**

| | | *v* | *A* | *D* | *α* | $A_{TS}$ | *NSE* |
|---|---|---|---|---|---|---|---|
| **E1** | *OTIS-P* | 0.0733 | 0.0364 | 0.0637 | 0.0006 | 0.0074 | **0.9984** |
| | *Identifiability analysis* | 0.0729 | 0.0370 | 0.0523 | 0.0014 | 0.0073 | 0.9977 |
| **E2** | *OTIS-P* | 0.2619 | 0.0481 | 0.0232 | 0.0091 | 0.0237 | 0.968 |
| | *Identifiability analysis* | 0.2732 | 0.0543 | 0.1111 | 0.0078 | 0.0182 | **0.9945** |
| **E3** | *OTIS-P* | 0.1236 | 0.0374 | 0.0705 | 0.0016 | 0.0066 | 0.9983 |
| | *Identifiability analysis* | 0.1220 | 0.0392 | 0.0758 | 0.0017 | 0.0057 | **0.9984** |
| **E4** | *OTIS-P* | 0.11 | 0.0356 | 0.0738 | 0.001 | 0.0064 | **0.9982** |
| | *Identifiability analysis* | 0.1111 | 0.0365 | 0.0678 | 0.0017 | 0.0056 | 0.9979 |
| **E5** | *OTIS-P* | 0.1774 | 0.0509 | 0.1151 | 0.0016 | 0.0077 | 0.9979 |

| | | | | | | | |
|---|---|---|---|---|---|---|---|
| | *Identifiability analysis* | 0.1863 | 0.0531 | 0.0927 | 0.0037 | 0.0076 | **0.999** |
| **E6** | *OTIS-P* | 0.1667 | 0.0479 | 0.09209 | 0.0025 | 0.0071 | **0.9989** |
| | *Identifiability analysis* | 0.1755 | 0.0479 | 0.0759 | 0.0051 | 0.0083 | 0.9985 |
| **E7** | *OTIS-P* | 0.275 | 0.0708 | 0.0113 | 0.0163 | 0.0261 | 0.9940 |
| | *Identifiability analysis* | 0.2894 | 0.0793 | 0.1189 | 0.0069 | 0.0159 | **0.9988** |
| **E8** | *OTIS-P* | 0.2292 | 0.0728 | 0.1752 | 0.0031 | 0.0122 | 0.9974 |
| | *Identifiability analysis* | 0.2440 | 0.0741 | 0.1440 | 0.0055 | 0.0140 | **0.9988** |
| **E9** | *OTIS-P* | 0.2444 | 0.0689 | 0.1475 | 0.0045 | 0.0128 | **0.9986** |
| | *Identifiability analysis* | 0.2550 | 0.0674 | 0.1259 | 0.0062 | 0.0142 | 0.9985 |
| **E10** | *OTIS-P* | 0.2245 | 0.0723 | 0.1778 | 0.0025 | 0.0104 | 0.9981 |
| | *Identifiability analysis* | 0.2503 | 0.0685 | 0.0984 | 0.0102 | 0.0176 | **0.9987** |
| **E11** | *OTIS-P* | 0.1250 | 0.0439 | 0.1566 | 0.0008 | 0.0071 | **0.9981** |
| | *Identifiability analysis* | 0.1206 | 0.0434 | 0.1566 | 0.0008 | 0.0076 | 0.9974 |
| **E12** | *OTIS-P* | 0.1279 | 0.0401 | 0.1475 | 0.0008 | 0.0059 | **0.9982** |
| | *Identifiability analysis* | 0.1226 | 0.0403 | 0.1488 | 0.0009 | 0.0052 | 0.9980 |
| **E13** | *OTIS-P* | NaN | NaN | NaN | NaN | NaN | NaN |
| | *Identifiability analysis* | 0.1210 | 0.0425 | 0.1557 | 0.0008 | 0.0082 | **0.9985** |
| **E14** | *OTIS-P* | 0.3438 | 0.0871 | 0.2694 | 0.0020 | 0.0162 | 0.9927 |
| | *Identifiability analysis* | 0.3486 | 0.0900 | 0.1984 | 0.0050 | 0.0130 | **0.9979** |
| **E15** | *OTIS-P* | 0.3235 | 0.0800 | 0.2775 | 0.0029 | 0.0139 | 0.9954 |
| | *Identifiability analysis* | 0.3506 | 0.0785 | 0.1816 | 0.0102 | 0.0177 | **0.9989** |
| **E16** | *OTIS-P* | 0.6707 | 0.1112 | 0.8761 | 0.0105 | 0.0226 | 0.9978 |
| | *Identifiability analysis* | 0.8239 | 0.0973 | 0.4599 | 0.0420 | 0.0403 | **0.9993** |
| **E17** | *OTIS-P* | NaN | NaN | NaN | NaN | NaN | NaN |
| | *Identifiability analysis* | 0.3858 | 0.1043 | 0.2582 | 0.0097 | 0.0283 | **0.9982** |
| **E18** | *OTIS-P* | 0.0591 | 0.0224 | 0.0336 | 0.0012 | 0.0082 | **0.9964** |
| | *Identifiability analysis* | 0.0583 | 0.0221 | 0.0346 | 0.0012 | 0.0082 | 0.9950 |
| **E19** | *OTIS-P* | NaN | NaN | NaN | NaN | NaN | NaN |
| | *Identifiability analysis* | 0.0480 | 0.0206 | 0.0181 | 0.0012 | 0.0074 | **0.9896** |
| **E20** | *OTIS-P* | 0.0308 | 0.0142 | 0.0226 | 0.0005 | 0.0068 | 0.9816 |
| | *Identifiability analysis* | 0.0296 | 0.0137 | 0.0200 | 0.0005 | 0.0074 | **0.9898** |
| **E21** | *OTIS-P* | 0.1418 | 0.0362 | 0.0924 | 0.0017 | 0.0060 | 0.9983 |
| | *Identifiability analysis* | 0.1518 | 0.0364 | 0.0716 | 0.0041 | 0.0066 | **0.999** |
| **E22** | *OTIS-P* | 0.1447 | 0.0359 | 0.1093 | 0.0012 | 0.0058 | 0.9979 |
| | *Identifiability analysis* | 0.1433 | 0.0371 | 0.1006 | 0.0016 | 0.0051 | **0.999** |
| **E23** | *OTIS-P* | 0.1447 | 0.0341 | 0.0957 | 0.0018 | 0.0055 | 0.9986 |
| | *Identifiability analysis* | 0.1444 | 0.0340 | 0.0910 | 0.0026 | 0.0055 | **0.9987** |
| **E24** | *OTIS-P* | 0.1410 | 0.0346 | 0.1025 | 0.0013 | 0.0054 | **0.9987** |
| | *Identifiability analysis* | 0.1414 | 0.0364 | 0.0943 | 0.0022 | 0.0044 | 0.9985 |
| **E25** | *OTIS-P* | 0.1392 | 0.0331 | 0.0734 | 0.0028 | 0.0063 | **0.999** |
| | *Identifiability analysis* | 0.1399 | 0.0334 | 0.0896 | 0.0022 | 0.0050 | 0.9982 |
| **E26** | *OTIS-P* | 0.1390 | 0.0339 | 0.0939 | 0.0015 | 0.0056 | 0.9987 |
| | *Identifiability analysis* | 0.1389 | 0.0341 | 0.0808 | 0.0022 | 0.0057 | **0.9991** |
| **E27** | *OTIS-P* | 0.1375 | 0.0341 | 0.0922 | 0.0015 | 0.0056 | **0.9987** |
| | *Identifiability analysis* | 0.1399 | 0.0335 | 0.0759 | 0.0025 | 0.0061 | 0.9985 |
| **E28** | *OTIS-P* | 0.1341 | 0.0319 | 0.1215 | 0.0008 | 0.0085 | 0.9941 |
| | *Identifiability analysis* | 0.1319 | 0.0337 | 0.1052 | 0.0011 | 0.0049 | **0.9971** |
| **E29** | *OTIS-P* | 0.0917 | 0.0307 | 0.0508 | 0.0009 | 0.0044 | 0.9985 |
| | *Identifiability analysis* | 0.0966 | 0.0311 | 0.0378 | 0.0023 | 0.0052 | **0.9986** |
| **E30** | *OTIS-P* | 0.0902 | 0.0286 | 0.0582 | 0.0006 | 0.0048 | 0.9956 |
| | *Identifiability analysis* | 0.0893 | 0.0305 | 0.0468 | 0.0011 | 0.0041 | **0.999** |
